# Methodological overview of systematic reviews to establish the evidence base for emergency general surgery

**DOI:** 10.1002/bjs.10476

**Published:** 2017-03-14

**Authors:** EMSurg Collaborators, J. Savović, N. Blencowe, J. Blazeby, Sean Strong, Noah Howes, K. Chalmers, K. Whale, J. Crichton, L. Gould, S. Kariyawasam, J. Mason, V. Pegna, S. Richards, C. Rowlands, D. Stevens

**Affiliations:** ^1^ Centre for Surgical ResearchSchool of Social and Community MedicineUniversity of BristolUK; ^2^ National Institute for Health Research Collaboration for Leadership in Applied Health Research and Care WestUniversity Hospitals Bristol NHS Foundation TrustBristolUK; ^3^ Division of Surgery, Head and NeckUniversity Hospitals Bristol NHS Foundation TrustBristolUK

## Abstract

**Background:**

The evidence for treatment decision‐making in emergency general surgery has not been summarized previously. The aim of this overview was to review the quantity and quality of systematic review evidence for the most common emergency surgical conditions.

**Methods:**

Systematic reviews of the most common conditions requiring unplanned admission and treatment managed by general surgeons were eligible for inclusion. The Centre for Reviews and Dissemination databases were searched to April 2014. The number and type (randomized or non‐randomized) of included studies and patients were extracted and summarized. The total number of unique studies was recorded for each condition. The nature of the interventions (surgical, non‐surgical invasive or non‐invasive) was documented. The quality of reviews was assessed using the AMSTAR checklist.

**Results:**

The 106 included reviews focused mainly on bowel conditions (42), appendicitis (40) and gallstone disease (17). Fifty‐one (48·1 per cent) included RCTs alone, 79 (74·5 per cent) included at least one RCT and 25 (23·6 per cent) summarized non‐randomized evidence alone. Reviews included 727 unique studies, of which 30·3 per cent were RCTs. Sixty‐five reviews compared different types of surgical intervention and 27 summarized trials of surgical versus non‐surgical interventions. Fifty‐seven reviews (53·8 per cent) were rated as low risk of bias.

**Conclusion:**

This overview of reviews highlights the need for more and better research in this field.

## Abstract 2

Evidence still limited

## Introduction

Unplanned, urgent and emergency surgery are terms used to describe the work undertaken by surgeons to manage a diverse and challenging group of pathologies linked by the need for unscheduled, non‐elective treatment. Attempts have been made to reach consensus regarding the primary conditions that represent emergency general surgical diagnoses, treated by general surgeons^1^. They may include upper and lower gastrointestinal tract pathology, hepatopancreatobiliary disease, appendicitis, anorectal soft tissue infections and abdominal wall hernias. These conditions comprise a substantial healthcare burden, accounting for 7 per cent of all US hospital admissions (equating to over 4 million inpatient encounters per year) and 50 per cent of a general surgeon's workload^2^, ^3^. In the UK, the most frequently performed emergency general surgical operations are incision and drainage of abscess, appendicectomy and cholecystectomy, whereas abdominal infections and bowel obstructions (with or without ischaemia) contribute the majority of operative workload^3^. A recent study from the USA^4^ found that the seven most frequent operations, which accounted for 80 per cent of emergency surgical procedures, were partial colectomy, small bowel resection, cholecystectomy, operative management of peptic ulcer disease, lysis of peritoneal adhesions, appendicectomy and laparotomy. Recent reports from the Royal College of Surgeons of England^3^, ^5^ found the delivery of unplanned and urgent general surgical care to be suboptimal with wide variations in outcomes, such as mortality, between hospitals^6^. Similarly, studies from the USA^7^, ^8^ have reported that outcomes of emergency and urgent abdominal surgery are variable and poorly measured. Reports highlighted the urgent need for well designed and conducted research to inform decision‐making, underpin national guidelines and influence health policy^7^.

The first step towards generating well designed research is to understand the current volume, quality and breadth of evidence. Evidence may take the form of primary research studies, ideally RCTs assessing effectiveness of treatments, health economic evaluations assessing cost‐effectiveness, or diagnostic studies comparing diagnostic procedures. Systematic reviews of evidence enable primary research studies investigating a common question to be summarized and assessed. Overviews of reviews are a recognized method of compiling and assessing the findings from multiple systematic reviews into one accessible and usable summary, which can then be used to identify evidence gaps and prioritize future research^9^, ^10^, ^11^, ^12^. The aim of this study, therefore, was to undertake an overview of systematic reviews in unplanned general surgery to obtain an understanding of the volume and quality of current evidence.

## Methods

This study is the first part of a larger body of work which includes: an overview of reviews of intervention studies; an overview of reviews of diagnostic studies; and a review of economic evaluation and cost‐effectiveness studies in unplanned and urgent general surgery. The review protocol is published in the PROSPERO systematic review register (CRD42015014198)^13^. Methods relating to the search strategies and study selection (which were common to all 3 parts of this work), and other methods specific to the intervention reviews, are described below.

### Inclusion criteria

Systematic reviews of interventions for patients with a condition of interest (see below), requiring unplanned and emergency treatment by general surgeons and published in English, were eligible. A systematic review was defined as one that made a documented attempt to identify studies addressing a research question of interest, with or without a statistical summary of included studies (meta‐analysis).

#### *Participants, conditions and interventions of interest*

Unplanned, urgent and emergency general surgery is a large clinical area. This overview therefore focused on the most common conditions managed by general surgeons in emergency settings, based on data from the Hospital Episode Statistics database in the UK^14^, and the Royal College of Surgeons report^3^. These are inflammatory, obstructive or ischaemic conditions affecting the small or large bowel; appendicitis; gallstone disease; peptic ulcer disease; anorectal soft tissue infections; and abdominal wall hernias. Reviews of acute trauma treated by general surgeons were excluded. All surgical, non‐surgical invasive (for example radiological and endoscopic) and non‐invasive (such as pharmacological) interventions were included as long as the condition was considered to be managed predominantly by general surgeons. For example, endoscopic or pharmacological interventions for bleeding peptic ulcer disease were excluded (being initially and primarily managed by gastroenterologists), whereas surgical interventions for the same condition were included. Care pathways and interventions for postoperative complications were excluded. Reviews reporting combined details of elective and urgent interventions were excluded unless the results could be extracted separately. Paediatric reviews (patients aged less than 16 years) were excluded. Also excluded were reviews where the sole purpose was to compare patients with different characteristics (such as different disease severity) all undergoing the same intervention.

### Search methods for identification of reviews

The following databases were searched from inception to April 2014: DARE (Database of Abstracts of Reviews of Effects), NHS EED (NHS Economic Evaluation Database) and HTA (Health Technology Assessments). In addition, the PROSPERO systematic reviews register was searched within the same time frame. Full search strategies are shown in *Appendix S1* (supporting information). No language restrictions were imposed at the search stage. Search hits were downloaded to a citation management program and duplicate records removed.

### Data collection and analysis

#### *Selection of reviews*

Titles and abstracts of search hits were screened independently by two reviewers with clinical expertise in the conditions of interest. Records with discrepant decisions were rescreened by a senior reviewer whose decision was final. More complex clinical queries were referred to senior members of the research team. Full papers were obtained for all relevant records (including those deemed unclear at the abstract stage) and assessed for inclusion by two reviewers independently, based on prespecified criteria (*Appendix S2*, supporting information). Disagreements were discussed and, if unresolved, a senior reviewer cast a final decision. When several versions of Cochrane reviews were identified, only the most recent was included. If there was more than one publication of an identical review (for example a Cochrane review and a journal version including the same papers), only the most detailed was included.

#### *Data extraction and management*

Data were extracted on a prespecified form that was piloted by two authors (*Appendix S3*, supporting information). For approximately one‐third of papers, data extraction was completed independently by two reviewers. As agreement was good, for subsequent reviews one reviewer extracted the data and another checked the extraction. Disagreements were resolved as described above.

The following information was extracted: basic bibliographic details; key review methods; start and end dates of the searches; types and number of included studies (RCTs, non‐randomized studies) and patients; the nature of interventions and comparators; and all synthesized outcomes. Where meta‐analyses were available, these were documented. Data were entered and stored in a custom‐made electronic database.

#### *Assessment of methodological quality of the systematic reviews*

Before the review began, three tools for quality assessment of reviews were piloted: Overview Quality Assessment Questionnaire (OQAQ)^15^, AMSTAR (a measurement tool with 11 items, specifically used to assess systematic reviews)^16^, and one proposed by Li and colleagues^12^. AMSTAR was selected as it was developed and validated specifically for the assessment of methodological quality of systematic reviews, and was the easiest to apply (Part G; *Appendix S3*, supporting information). However, it does not provide guidance on how to integrate the 11 items into an overall risk‐of‐bias judgement. A previously described method was therefore used^17^ in which reviews were considered to be at low risk of bias, and thus of high methodological quality, if the following four items were satisfied: a comprehensive literature search; assessment of the scientific quality of the included studies; appropriate use of quality assessments in formulating review conclusions; and appropriate use of methods to combine findings. Reviews failing to meet one or more of these criteria were considered to be at high risk of bias. If insufficient details were provided to permit judgement on one or more items, a review was deemed to be at unclear risk^17^. The AMSTAR criteria were applied independently by two reviewers and disagreements resolved through discussion.

### Data synthesis

Included reviews were summarized descriptively by each condition, including the number of reviews, and the number and type of included studies (RCTs or non‐randomized studies). Conditions of the small and large bowel are heterogeneous, but they were summarized together because in the emergency setting these conditions (such as bowel obstruction, colitis and diverticulitis) are frequently managed by general surgeons. The number of included patients was documented for each review. To examine the overall volume of evidence, the total number of unique primary studies and their design were recorded for each condition (thus avoiding double‐counting of the same studies cited in multiple reviews). The nature of the interventions (surgical, non‐surgical invasive (endoscopic or radiological) or non‐invasive) was documented and mapped by study design to identify evidence gaps. Details about outcomes of specific reviews are not reported here and will be the focus of subsequent disease‐specific publications.

## Results

**Figure 1:**
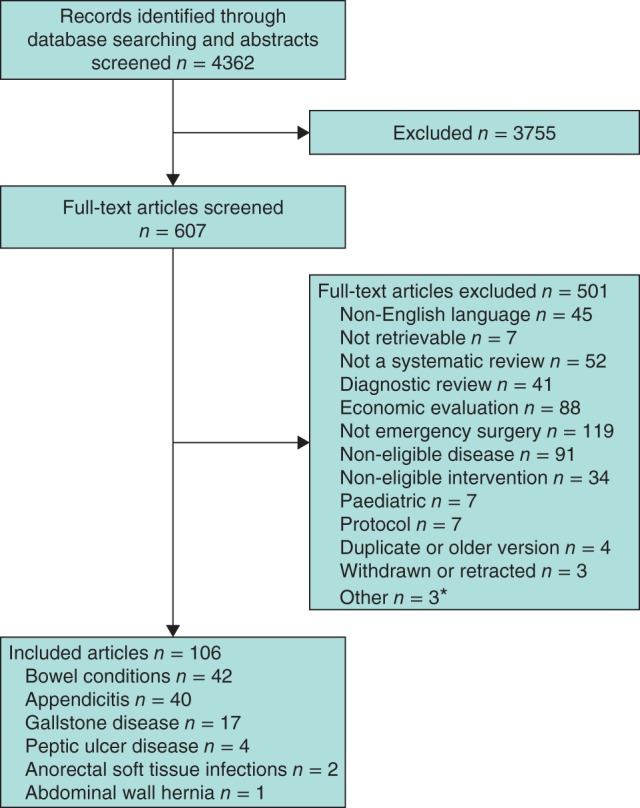
PRISMA flow chart for the overview. *Comparison of patients with different disease severity, all undergoing the same intervention (1) and review not focused on the main intervention for treating the eligible condition (2)

Searches identified 4362 hits; 607 were considered potentially relevant and, of these, 555 were obtained and read in full. The remaining 52 papers were not assessed; seven could not be obtained (withdrawn, superseded or could not be obtained via an interlibrary loan or from the authors/publishers) and 45 were not written in English. A total of 106 reviews were included (*Fig*. 1). A full list of excluded studies with reasons is available in *Appendix S4* (supporting information).

### Characteristics of included reviews

The included 106 reviews focused on bowel conditions (42 reviews)^18^, ^19^, ^20^, ^21^, ^22^, ^23^, ^24^, ^25^, ^26^, ^27^, ^28^, ^29^, ^30^, ^31^, ^32^, ^33^, ^34^, ^35^, ^36^, ^37^, ^38^, ^39^, ^40^, ^41^, ^42^, ^43^, ^44^, ^45^, ^46^, ^47^, ^48^, ^49^, ^50^, ^51^, ^52^, ^53^, ^54^, ^55^, ^56^, ^57^, ^58^, ^59^, appendicitis (40)^60^, ^61^, ^62^, ^63^, ^64^, ^65^, ^66^, ^67^, ^68^, ^69^, ^70^, ^71^, ^72^, ^73^, ^74^, ^75^, ^76^, ^77^, ^78^, ^79^, ^80^, ^81^, ^82^, ^83^, ^84^, ^85^, ^86^, ^87^, ^88^, ^89^, ^90^, ^91^, ^92^, ^93^, ^94^, ^95^, ^96^, ^97^, ^98^, ^99^, gallstone disease (17)^100^, ^101^, ^102^, ^103^, ^104^, ^105^, ^106^, ^107^, ^108^, ^109^, ^110^, ^111^, ^112^, ^113^, ^114^, ^115^, ^116^, peptic ulcer disease (4)^117^, ^118^, ^119^, ^120^, anorectal soft tissue infections (2)^121^, ^122^ and abdominal wall hernias (1)^123^. The reviews of bowel conditions encompassed a diverse group of conditions that included bowel obstructions, colitis, ischaemia and diverticulitis. The 106 reviews included a median of 8 studies (i.q.r. 5–15), although two reviews^33^, ^66^ were empty (no eligible studies were identified). The 106 reviews summarized results from 727 unique papers, of which 220 were RCTs (30·3 per cent). Seventy‐nine reviews (74·5 per cent) included at least one RCT, 51 (48·1 per cent) included exclusively RCTs and 25 (23·6 per cent) summarized solely non‐randomized evidence. The highest number of included RCTs was in reviews of appendicitis (106 of 176 included studies in this category); there were just four unique RCTs in peptic ulcer disease, and none in abdominal wall hernia. The median number of patients included in the reviews varied between conditions, from eight for abdominal wall hernia to 1235 for appendicitis. The median number of patients in the RCTs included in the reviews also varied, from zero for abdominal wall hernia to 701 for appendicitis. Detailed descriptions of the systematic reviews by condition are provided in *Table* 1.

**Table 1:** Descriptions of systematic reviews by condition of interest

	No. of reviews	No. of studies in reviews*	No. of RCTs in reviews	No. of patients in reviews	No. of patients in included RCTs
Bowel conditions	42	9 (5–16; 0–98)	2 (0–4; 0–9)	509 (233–878; 0–3975)	78 (0–315; 0–1074)
Appendicitis	40	10 (5–17; 0–57)	5 (1–15; 0–56)	1235 (744–2277; 0–57 851)	701 (9–1381; 0–5896)
Gallstone disease	17	5 (3–10; 1–53)	3 (1–6; 0–28)	488 (272–878; 51–3659)	388 (63–595; 0–3659)
Peptic ulcer disease	4	9 (3–15; 3–15)	3 (2–4; 2–4)	487 (296–999; 289–1113)	252 (178–309; 166–315)
Anorectal soft tissue infection	2	6 (5–6; 5–6)	6 (5–6; 5–6)	442 (405–479; 405–479)	442 (405–479; 405–479)
Abdominal wall hernia	1	8	0	8	0

Values are median (i.q.r.; range).

* Includes all types of study design.

### Interventions summarized in the reviews

Most reviews summarized studies comparing different types of surgical treatment (65 reviews, 165 unique RCTs) (*Table* 2). For some conditions (peptic ulcer disease, anorectal soft tissue infections and abdominal wall hernia) no other types of review were identified. Just seven reviews (including 21 unique RCTs) compared different types of non‐invasive treatment, all for bowel conditions managed by general surgeons. Reviews of non‐surgical invasive treatments (such as endoscopic or radiological interventions) were identified only for bowel and gallstone disease, and few reviews examined the evidence for surgical *versus* non‐surgical treatment (whether invasive or non‐invasive).

**Table 2:** Characteristics of included reviews and numbers of RCTs by types of intervention

	Types of intervention compared					
	Surgical treatments	Surgery *versus* non‐surgical invasive treatments	Surgery *versus* non‐invasive treatments	Non‐surgical invasive *versus* non‐invasive treatments	Non‐surgical invasive treatments	Non‐invasive treatments
Bowel conditions (42 reviews, 47 RCTs*)	14 reviews	14 reviews	4 reviews	1 review†	2 reviews	7 reviews
	8 RCTs	12 RCTs	0 RCTs	0 RCTs	8 RCTs	21 RCTs
Appendicitis (40 reviews, 106 RCTs)	33 reviews	0	7 reviews	0	0	0
	100 RCTs		6 RCTs			
Gallstone disease (17 reviews, 57 RCTs)	11 reviews	2 reviews	0	4 reviews	0	0
	47 RCTs	3 RCTs‡		7 RCTs		
Peptic ulcer disease (4 reviews, 4 RCTs)	4 reviews	0	0	0	0	0
	4 RCTs					
Anorectal soft tissue infection (2 reviews, 6 RCTs)	2 reviews	0	0	0	0	0
	6 RCTs					
Abdominal wall hernia (1 review, 0 RCTs)	1 review§	0	0	0	0	0
	0 RCTs					
Total (106 reviews, 220 RCTs)	65 reviews	16 reviews	11 reviews	5 reviews	2 reviews	7 reviews
	165 RCTs	15 RCTs	6 RCTs	7 RCTs	8 RCTs	21 RCTs

Number of reviews and number of unique RCTs included in reviews are shown. Non‐surgical invasive treatments include all endoscopic or radiological procedures; non‐invasive treatments include all pharmacological interventions (such as antibiotics, intravenous fluid regimens).

* Two RCTs were included in multiple reviews across two different intervention comparisons (surgical *versus* surgical treatments and surgical *versus* non‐surgical invasive treatments) and were therefore included in both intervention categories.

† Review included 25 non‐randomized case series (no comparator) reporting outcomes from a total of 315 patients^.^

‡ Surgery *versus* radiological treatments.

§ Review included 17 non‐randomized case series studies (no comparator) reporting outcomes from a total of 28 patients, of which only 8 single‐patient case reports were in emergency settings.

### Methodological quality of reviews

The methodological quality of the reviews was variable, meeting between one and ten AMSTAR items (median 7, i.q.r. 5–9). Just over half (57, 53·8 per cent) met all four of the key AMSTAR items and were thus considered at low risk of bias. Common reasons for being assessed as high risk were failure to apply quality assessments of included studies to appropriately formulating review conclusions (31, 29·2 per cent), not assessing the quality of included studies (28, 26·4 per cent), not conducting a comprehensive literature search (16, 15·1 per cent), and using inappropriate methods to combine the studies statistically, or combining when it was not appropriate to do so (30, 28·3 per cent). The majority of reviews (85 of 106, 80·2 per cent) included one or more meta‐analyses. Most reviews (11 of 17) about gallstone disease were rated as low risk of bias, as were the two reviews of anorectal soft tissue infection; the sole hernia review was rated as high risk of bias. Detailed assessments of methodological quality are shown in *Table* 3.

**Table 3:** Critical appraisal of systematic reviews by condition of interest

AMSTAR criteria	No. of reviews that met specified AMSTAR criteria					
	Bowel conditions (*n =* 42)	Appendicitis (*n =* 40)	Gallstone disease (*n =* 17)	Peptic ulcer disease (*n =* 4)	Anorectal soft tissue infection (*n =* 2)	Abdominal wall hernia (*n =* 1)
*A priori* design	28	29	15	2	2	0
Duplicate study selection and data extraction	32	32	14	3	2	0
Adequate literature search†	39	29	16	3	2	1
Inclusion not restricted by publication status	21	20	5	2	1	0
Included and excluded studies listed	15	12	10	1	0	0
Details of included studies provided	29	31	14	4	2	1
Scientific quality of included studies assessed†	31	29	14	2	2	0
Conclusions appropriate based on study quality†	30	27	14	2	2	0
Appropriate methods to combine study results†	26	35	9	4	2	0
Publication bias assessed	16	23	10	2	1	0
Conflict of interest included	8	10	1	4	0	0
Overall risk of bias rated low‡	22	20	11	2	2	0
No. of AMSTAR criteria met (of 11)*	6 (1–10)	7 (1–10)	8 (2–10)	6 (3–10)	10 (10–10)	2

* Values are median (range).

† Key AMSTAR criteria for assessment of overall risk of bias.

‡ Low risk was assigned to reviews that fulfilled all four key criteria (comprehensive literature search was performed; the scientific quality of the included studies was assessed; these quality assessments were then used appropriately in formulating review conclusions; and the methods used to combine the findings were appropriate).

## Discussion

This synthesis included 106 systematic reviews summarizing evidence for unplanned, urgent and emergency general surgery. Although good numbers of reviews were available for bowel conditions and appendicitis (42 and 40 respectively), the summarized evidence for the treatment of emergency hernias and anorectal soft tissue infections was sparse. Evidence from RCTs was most prevalent in reviews of gallstone disease and appendicitis, and least prevalent in bowel conditions. Similarly, gallstone disease and appendicitis had the highest number of large RCTs (over 500 participants). The quality of included reviews was variable, with just over half being rated as low risk of bias. There was a paucity of RCTs comparing surgery and non‐surgical interventions (whether these were invasive or non‐invasive). It is recommended that future research is prioritized in the areas where there are limited numbers of well designed and conducted RCTs and systematic reviews^12^, ^124^.

Understanding the state of current evidence and areas where it is lacking is a valuable exercise to map the evidence base, and inform commissioning of primary and secondary research. It also highlights the need to provide educational research opportunities for general surgeons themselves. The observed lack of evidence may be representative of an unfamiliarity with trials, collaborative working and the need for high‐quality evidence. Provision of facilities for research training and opportunities to participate in well designed studies is therefore recommended. Over the past decade this issue has partially been addressed by the Royal College of Surgeons surgical trials initiative^125^, ^126^. Several multicentre trainee‐led studies^127^, ^128^, ^129^, ^130^, ^131^ have been successfully designed, conducted and reported. Once these trainees complete their surgical training the opportunity for more research will escalate, and the collective experiences and knowledge will equip the surgical community to undertake more difficult trials in the emergency general surgical setting.

Overviews of evidence are an important tool for prioritization of any future research^12^, ^124^. Overviews of systematic reviews may provide opportunities for considerable cost savings if their findings are used to focus future research efforts and reduce research waste (for example by identifying that a question has already been answered and does not require further research). The quality of primary studies and their syntheses should be considered in this process. Historically, RCTs have not been undertaken readily in surgery because of methodological issues with blinding and intervention complexity. These are compounded in the emergency surgical setting, with particular challenges to recruitment and data collection^132^. Although little is known about how to optimize data collection in this setting, a recent study^133^ has examined the feasibility of collecting patient‐reported outcome data during unplanned hospital admissions. It found that, with specific research support during the working week, good baseline response rates to questionnaires could be achieved.

Another area for further work is to identify whether there are primary RCTs in the areas where no reviews were identified. It is possible that RCTs have been undertaken but not yet reviewed. In some areas the reason for a lack of reviews will be the lack of primary studies, and future work should focus on conducting good‐quality RCTs to provide answers to clinical questions.

Non‐English‐language studies were excluded for logistical reasons and this means that some reviews were likely missed. However, multiple reviews were identified for most topics, usually with overlapping trials. It is thus reasonable to assume that most topics for which reviews are conducted will have at least one review published in English. Some reviews had been published multiple times but the duplicate publications were not always identical. Duplicate publications were removed where possible, but when the two publications differed, both were included.

The evidence base for the care of some patients requiring emergency treatment by general surgeons is poor. Evidence for emergency hernia repair and treatment of anorectal abscess is currently particularly sparse. There is also a lack of comparative evidence to inform clinical decisions regarding invasive *versus* non‐invasive interventions in this setting, where patients may be high risk and may benefit from less invasive treatment options. Although these types of trial may be particularly difficult to undertake, they are key to influencing practice and should be encouraged. It is therefore recommended that focused and better multicentre studies are undertaken.

## Collaborators

J. Savović (J.S.)*^,^ †, N. Blencowe (N.S.B.)* and J. Blazeby (J.M.B.)*^,^‡, designed the study, wrote the protocol and together led the project, which was conceived by J.M.B.* J.S. and N.S.B. designed the screening and data extraction forms. N.S.B. developed systematic literature searches. J.S. oversaw the review process, resolved discrepancies, constructed tables and figures and wrote the main body of the manuscript. N.S.B., Sean Strong (S.S.)* and Noah Howes (N.H.)* screened abstracts and full papers, resolved discrepancies and checked data extractions. N.S.B. and S.S. made substantial contributions to the writing of the manuscript and tables. J.S., K. Chalmers (K.C.)* and K. Whale* designed the database for data collection and carried out full paper screening and data extraction. K.C. also completed data analyses, managed the database for the review and contributed to the manuscript. N.H., J. Crichton*, L. Gould*, S. Kariyawasam*, J. Mason*, V. Pegna*, S. Richards*, C. Rowlands* and D. Stevens* wrote sections of the study protocol and contributed to the study design, screened abstracts and full papers, extracted data, resolved discrepancies and checked data extractions. All authors read and approved the final manuscript.

*Centre for Surgical Research, School of Social and Community Medicine, University of Bristol, UK; †National Institute for Health Research Collaboration for Leadership in Applied Health Research and Care West, University Hospitals Bristol NHS Foundation Trust, Bristol, UK; ‡Division of Surgery, Head and Neck, University Hospitals Bristol NHS Foundation Trust, Bristol, UK.

**Supporting information**

Additional supporting information may be found in the online version of this article:

**Appendix S1** Search strategy (Word document)

**Appendix S2** Study selection form (Word document)

**Appendix S3** Data extraction form (Word document)

**Appendix S4** List of excluded studies with reasons (Word document)

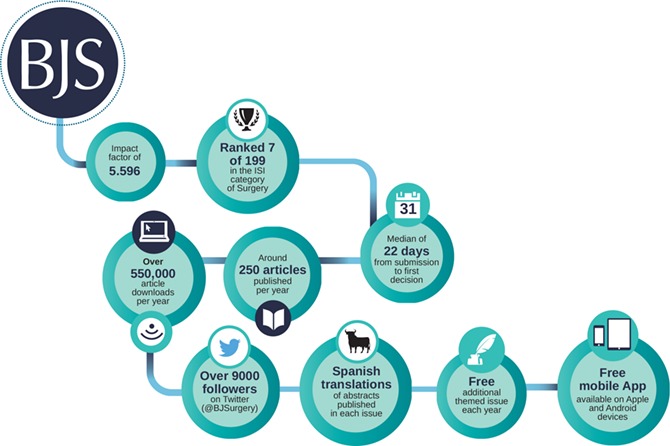

## Supporting information


**Appendix S1** Search strategy
**Appendix S2** Study selection form
**Appendix S3** Data extraction form
**Appendix S4** List of excluded studies with reasons
