# Supplementary material for: Methodological overview of systematic reviews to establish the evidence base for emergency general surgery
Source: Br J Surg. 2017 Mar 14;104(5):513–24. doi: 10.1002/bjs.10476 (PMC5363346; doi:10.1002/bjs.10476)
Supplement: Supplementary file 1 — Appendix S1 Search strategy Appendix S2 Study selection form Appendix S3 Data extraction form Appendix S4 List of excluded studies with reasons [file BJS-104-513-s001.docx]

**BJS10476**

**Methodological overview of systematic reviews to establish the evidence base for emergency general surgery**

**EMSurg Collaborators**

**Appendix S1** Search strategy

Search Strategies for Centre for Reviews and Dissemination (CRD) databases

Appendicitis

1 MeSH DESCRIPTOR Appendicitis EXPLODE ALL TREES

2 MeSH DESCRIPTOR Appendix EXPLODE ALL TREES

3 MeSH DESCRIPTOR Appendectomy EXPLODE ALL TREES

4 (appendicitis)

5 (appendectomy)

6 (Appendicectomy)

7 ((appendix) AND (rupture* or perforat*))

8 #1 OR #2 OR #3 OR #4 OR #5 OR #6 OR #7

Abscess

1 MeSH DESCRIPTOR Anus Diseases EXPLODE ALL TREES

2 MeSH DESCRIPTOR Anal Canal EXPLODE ALL TREES

3 #1 OR #2

4 MeSH DESCRIPTOR Abscess EXPLODE ALL TREES

5 MeSH DESCRIPTOR fistula

6 MeSH DESCRIPTOR Suppuration

7 MeSH DESCRIPTOR Sepsis

8 #4 OR #5 OR #6 OR #7

9 #3 AND #8

10 fistula-in -ano

11 (anus or anal or perianal* or peri-anal* or perirectal* or peri-rectal* or anoperineal* ) OR (anorectal* or ano-rectal*)

12 (abscess* or fistula* or sepsis*)

13 #11 AND #12

14 #9 OR #10 OR #13

Gallbladder

1 MeSH DESCRIPTOR Biliary Tract Surgical Procedures EXPLODE ALL TREES

2 MeSH DESCRIPTOR Biliary Tract EXPLODE ALL TREES

3 MeSH DESCRIPTOR Biliary Tract Diseases EXPLODE ALL TREES

4 MeSH DESCRIPTOR Pancreatitis EXPLODE ALL TREES

5 #1 OR #2 OR #3 OR #4

6 (Cholecystectom*)

7 (Cholecystostom*)

8 (Cholecystolithiasis)

9 (Choledocholithiasis)

10 (Cholecystitis)

11 (Cholelithiasis)

12 (pancreatitis)

13 (gallstone*)

14 (gall stone*)

15 (biliary colic)

16 #6 OR #7 OR #8 OR #9 OR #10 OR #11 OR #12 OR #13 OR #14 OR #15

17 #5 OR #16

Hernia and bowel

1 MeSH DESCRIPTOR Hernia, Abdominal EXPLODE ALL TREES

2 MeSH DESCRIPTOR Hernia, Obturator

3 MeSH DESCRIPTOR Herniorrhaphy

4 #1 OR #2 OR #3

5 (herni*) AND ((inguinal* or femoral* or ventral* or obturator* or umbilical*) )

6 (herniorrhaphy)

7 #5 OR #6

8 #4 OR #7

1 MeSH DESCRIPTOR Colonic Diseases EXPLODE ALL TREES

2 MeSH DESCRIPTOR Intestinal Obstruction EXPLODE ALL TREES

3 MeSH DESCRIPTOR Abdomen, Acute

4 MeSH DESCRIPTOR Digestive System Surgical Procedures

5 MeSH DESCRIPTOR Colectomy EXPLODE ALL TREES

6 MeSH DESCRIPTOR Enterostomy EXPLODE ALL TREES

7 MeSH DESCRIPTOR Intestinal Perforation

8 MeSH DESCRIPTOR Colonoscopy EXPLODE ALL TREES

9 MeSH DESCRIPTOR Surgical Stomas

10 MeSH DESCRIPTOR Rectal Diseases EXPLODE ALL TREES

11 MeSH DESCRIPTOR Cecal Diseases

12 MeSH DESCRIPTOR Cecal Neoplasms

13 #1 OR #2 OR #3 OR #4 OR #5 OR #6 OR #7 OR #8 OR #9 OR #10 OR #11 OR #12

14 (acute abdomen)

15 (colostomy)

16 (ileostomy)

17 (diverticulitis)

18 (enterostomy)

19 (colonoscopy)

20 (colon or rectum or rectal or intestine or intestinal or duodenum or duodenal or bowel or diverticular):TI

21 (colectomy)

22 (crohn's or crohns)

23 (colorectal):TI

24 #14 OR #15 OR #16 OR #17 OR #18 OR #19 OR #20 OR #21 OR #22 OR #23

25 #13 OR #24

Stomach and duodenum

1 MeSH DESCRIPTOR Peptic Ulcer EXPLODE ALL TREES

2 MeSH DESCRIPTOR Gastrointestinal Hemorrhage EXPLODE ALL TREES

3 MeSH DESCRIPTOR Digestive System Surgical Procedures

4 MeSH DESCRIPTOR Gastrectomy

5 #1 OR #2 OR #3 OR #4

6 (gastric ulcer*)

7 (peptic ulcer*)

8 (duodenal ulcer*)

9 (stomach ulcer*)

10 (gastrectomy)

11 (gastric or stomach or duoden* or gastrointestinal):TI AND (haemorrhag* or hemorrhag* or bleed*)

12 #6 OR #7 OR #8 OR #9 OR #10 OR #11

13 #5 OR #12

**Appendix S2** Study selection form

**Overview of systematic reviews in emergency surgery**

**Paper inclusion/exclusion form**

**Citation details**

First author .................................................. Publication year............................

**Citation ID** [essential] .......................................

Assessor name ............................................. Date .......... / ............. /................

**Study selection criteria**

[Please circle Yes or No. If the paper is not in English circle “Non-English paper” below – no need to assess Q 1 to 5.]

1. Is this paper a systematic review and/or economic evaluation? Yes No Unsure

2. Does the paper report on patients with emergency conditions? Yes No Unsure

**Yes** if review includes data on both elective and emergency patients, but data for emergency patients available separately.

3. Does the paper report on relevant condition/disease sites? Yes No Unsure

Gallbladder, appendix, bowel, stomach/duodenum, perianal abscess, hernia

4. Does the paper report on interventions or diagnostics? Yes No Unsure

Intervention may include drugs, surgery, devices, radiology, physiotherapy etc., but not organization of care.

5. Does the paper include adult patients? Yes No Unsure

**Yes** if it includes adults only, or mixture of adults, adolescents / children. **No** if paediatric only (& tick box below).

**Status of study** [Please circle]

*If the answer to all 5 questions are ‘yes’, include the study.*

**Excluded** / **Included** / **Pending** / **Non-English paper**

If excluded, main reason for excluding: ..................

Enter **number** of the first question you answered No to, e.g. if the study did not report on treatments or diagnostic enter 4.

This paper reports on the following condition(s): [Tick all that apply]

Abscess 🞏 Appendix 🞏 Bowel 🞏

Gallbladder 🞏 Stomach/duodenum 🞏 Hernia 🞏

This paper reports on: [Tick all that apply]

Interventions/treatments 🞏 Paediatric patients 🞏

Diagnostic tests 🞏

Economic evaluations 🞏 **Decision entered in database** **🞏**

**Appendix S3** Data extraction form

**Evidence in non-trauma emergency surgery:**

**A systematic review of systematic reviews of interventions**

**A Unified Data Extraction Form for Multiple Topics**

**Review ID No....................... First author................................................. Year..................**

**Journal.....................................................................................................................................................**

**0.1 In which one of the six overview topics/sites is this intervention review paper included?**

[Tick only one. If the paper reports on more than one of the 6 topics, then complete a separate extraction form for each topic. If this is not possible contact JS, JMB and NB to discuss a possible protocol revision.]

Abscess 🞏 Appendix 🞏 Bowel 🞏

Gallbladder 🞏 Stomach/duodenum 🞏 Hernia 🞏

**0.2 Extracted by ....................................................... 0.3 Date extracted ........................................**

**0.4 Checked by.......................................................... 0.5 Date checked .........................................**

**Discrepancies resolved and ready to enter?** 🞏 [To be ticked by person who checked]

**Part A: General characteristics of the review and types of included studies**

**A1 Start date of the search**...................................... **A2 End date of the search**.......................................

[month and year. If the start date differs for each database, state the oldest for start date.]

**Publication year of the:**

**A3 Oldest included study** ...................................... **A4 Most recent study**.........................................

**A5 Did the authors search the following databases?** [Tick all that apply]

^a^MEDLINE □ ^c^ISI Web of Science □

^b^EMBASE □ ^d^Cochrane CENTRAL/CCTR □

^e^Others □

^f^ Specify if others....................................................................................................................................

**A6 Did the authors use any supplementary sources to identify relevant studies?** [Tick all that apply]

^a^Conference proceedings □ ^e^Contacted manufacturers □

^b^Consulted reviews □ ^f^Theses/dissertations □

^c^Consulted experts □ ^g^Other grey literature □

^d^Checked references of included studies □ ^h^Other sources □

^i^Specify if other sources.............................................................................................................................

**A7 Did the review authors restrict the inclusion of eligible studies by study design?**

[E.g.: they only included RCTs; only RCTs, CCTs and cohorts; they only included studies in which 2 interventions were compared; or they included all study types except case-series, or similar descriptions. Look for evidence of this in the methods section, eligible studies section (if available), or in exclusion and inclusion criteria.]

Yes □ No □ Unsure/unclear □

**A7a** Explanation/comment (if needed):............................................................................................................

.............................................................................................................................................................................

**A8-A11 Types of included study and number of each**

[Tick all that apply and state the number of studies and patients for each type]

No of studies: No of patients:

^A8^ RCT □ ^a^ ....................... ^b^ ........................ ^c^

^A9^ Cohort study □ ^a^ ....................... ^b^ ........................ ^c^

^A10^ Case-series □ ^a^ ...................... ^b^ ........................ ^c^

^A11^ Other □ ^a^ ...................... ^b^ ........................ ^c^

^A11d^ Specify if other........................................................................................................................

**A12 Does the review include at least one meta-analysis?**

[By meta-analysis we mean any numerically pooled data, it does not have to have forest plots.]

Yes □ No □ Unsure □

**A13 Conclusions of the review (from the abstract) to be pasted directly into the database.**

[No extraction on paper is required for this item. Data abstractor should highlight the conclusion in the abstract clearly with the highlighter pen, so that the person doing data entry knows what to paste from the pdf – data enterer may be a non-expert.]

**Part B: Participants**

**B1 In terms of the age of the included patients, which category best describes patients included in the review?** [Tick one box]

Adults only □ Adults and children (incl. adolescents) □

Adults and adolescents □ Not stated □

Adolescents and/or children * □ Other □

^B1a^ If other, specify…………………………………………………………….....................................................................

[* The review should not have been included if it did not include any adults]

**B2 Did the review authors clearly define the characteristics of patients (and especially patients’ medical conditions) that are eligible for inclusion in the review?**

Yes □ No □ Unsure/unclear □

^B2a^ Explanation/comment (if needed):................................................................................................................

.............................................................................................................................................................................

**B3 List the inclusion criteria relating to patients and their conditions defined in this review:**

……………………………………………………………......................................................................................................

……………………………………………………………......................................................................................................

.....................................................................................................................................................................

.....................................................................................................................................................................

.....................................................................................................................................................................

**B4 List the exclusion criteria relation to patients and their conditions defined in this review:**

……………………………………………………………......................................................................................................

……………………………………………………………......................................................................................................

....................................................................................................................................................................

.....................................................................................................................................................................

.....................................................................................................................................................................

**Part C: Interventions**

**C1 Did the review authors clearly state which interventions are eligible for inclusion in the review?**

Yes □ No □ Unsure/unclear □

Explanation/comment (if needed):..................................................................................................................

..........................................................................................................................................................................

**C2 What is the nature of the primary intervention(s) of interest in this review?**

[If review is assessing a variety of interventions and isn’t clear which is the intervention of primary interest for the review, can the interventions be described as a group of interventions (e.g. all surgical vs non-surgical)? If still unclear, then we will arbitrarily describe here the first intervention mentioned in the title or abstract of the review. Tick one box only.]

Surgical □ Radiological □

Endoscopic □ Pharmacological □

Expectant management □ Combination of 2 or more types □

Other □ Not stated □

^C.2a^ If other, specify…………………………………………………………….........................................................................

…………………………………………………………….......................................................................................................

**C3 Brief description/name of the primary intervention(s) of interest in this review:**

[Keep it brief, database allows 255 characters only for this field, enter more details below]

……………………………………………………………......................................................................................................

……………………………………………………………......................................................................................................

.....................................................................................................................................................................

**C4 Please provide a more detailed description of the intervention, if available.**

[No extraction on paper is required for this item. Data abstractor should highlight the details of the intervention in the text clearly with a highlighter pen, so that the person doing data entry knows what to paste from the pdf – data enterer may be a non-expert.]

No further description provided □

**C5 What is the nature of the comparative intervention(s) assessed in this review?**

[Comparative interventions are those that review authors have chosen to compare against their primary intervention of interest, often these will be the more standard interventions, older interventions or usual care, but not always, sometimes it can be a bit arbitrary. Tick one box only.]

Surgical □ Radiological □

Endoscopic □ Pharmacological □

Expectant management □ Combination of 2 or more types □

Other □ Not stated □

^C.5a^ If other, specify…………………………………………………………….........................................................................

…………………………………………………………….......................................................................................................

**C6 Brief description/name of the comparative intervention(s):**

[Keep it brief, database allows 255 characters only for this field, enter more details below]

……………………………………………………………......................................................................................................

……………………………………………………………......................................................................................................

.....................................................................................................................................................................

**C7 Please provide a more detailed description of the comparative intervention, if available.**

[No extraction on paper is required for this item. Data abstractor should highlight the details of the intervention in the text clearly with a highlighter pen, so that the person doing data entry knows what to paste from the pdf – data enterer may be a non-expert.]

No further description provided □

**Part D: Outcomes**

**D1 Were the outcomes of interest for the systematic review defined *a priori*?**

Yes □ No □ Unsure/unclear □

^D1a^ Explanation/comment (if needed):................................................................................................................

..........................................................................................................................................................................

**D2 Did the review authors clearly state the primary outcome(s) of the review?**

Yes □ No □ Unsure/unclear □

^D2a^ Explanation/comment (if needed):................................................................................................................

..........................................................................................................................................................................

**Descriptions and codes for outcome domains**

[Choose the appropriate code for each outcome and enter it in the appropriate box in the next table]

| **Domain code** | **Outcome domain** | **Definition** |
| --- | --- | --- |
| **1** | Mortality | Outcomes related to short and long-term survival/death rates and cause of death |
| **2** | Complications | Forms of short and long-term postoperative morbidity |
| **3** | Peri-operative technical outcomes | Outcomes recorded directly in the operating theatre (e.g. operation time, blood loss) |
| **4** | Treatment pathway outcomes | Outcomes related to the flow of patients through the healthcare system (e.g. hospital stay, readmission) |
| **5** | Patient-reported outcomes | Outcomes reported by patients themselves |
| **6** | Symptoms / function | Outcomes assessed by an observer (usually a clinician) |
| **7** | Pathology / histology / laboratory findings | Histopathology, microbiology findings or results of laboratory tests carried out by assessor not otherwise involved in study participant care (e.g. blood or urine tests, biochemistry, microbiology etc.) |
| **8**  **8a**  **8b** | Cost / resources:  Cost of intervention  Cost of other resource use | Any measures of resource use expressed in monetary terms (e.g. cost of equipment). These can be the cost of the intervention itself or cost for the associated use of other resources. |
| **9** | Composite outcomes | An outcome that is a combination of outcomes analysed together, (e.g. death **or** disability) |
| **10** | Other outcomes | Outcome type that don’t fit any of the above categories |

**Descriptions and codes for study designs** (of studies included in reviews) [Applicable to part 5 - Results table]

1= RCTs only, 2=Comparative NRS only, 3=RCTs and comparative NRS pooled together, 4=Non-comparative studies (e.g. case series).

**Important**: Always extract separate results for RCTs and NRS is they are available. You don’t need to extract both separate and combined if separate is available. Extract combined if that’s the only result available.

**Reported outcomes and their definitions** [List all outcomes reported in the review for which there is an extractable result. Print and attach additional sheets of paper if needed]

| **Outcome number**  ^D3^ [assign each outcome unique No] | **Outcome name**  ^D4^  [verbatim as in the review] | **Domain code** ^D5^ [choose from table above] | **Meta-analysis?**  ^D7^  ***** | **Defined?**   ^D8^ [Yes / No / Partially] | **If defined, please write definition verbatim**  ^D10.^  [as reported in the review, not in each included study. If the review states that outcomes were as described in individual studies, then write that.] | Primary or secondary outcome of review?  ^D11^ [circle or cross-out] |
| --- | --- | --- | --- | --- | --- | --- |
|  |  |  |  |  |  | Primary  Secondary  Not stated |
|  |  |  |  |  |  | Primary  Secondary  Not stated |
|  |  |  |  |  |  | Primary  Secondary  Not stated |
|  |  |  |  |  |  | Primary  Secondary  Not stated |
|  |  |  |  |  |  | Primary  Secondary  Not stated |
|  |  |  |  |  |  | Primary  Secondary  Not stated |

* Was a meta-analysis performed for this outcome?

**Patient-reported outcomes – additional details** [Please list all PROs available in the review for which there is an extractable result. Print and attach another page if needed.]

List the measured PROs and any ad-hoc questions. Write ad hoc questions verbatim:

| **Outcome number**  ^D3^  [same as in table above] | **Outcome name**  ^D4^ [must be the same as in above table for all outcomes in domain 5, verbatim] | **What instrument was used?**  ^D12^ [if a well know Q-re, e.g. SF36] | **Validated?**  **Y/N/unclear**  ^D13^ | **If not a well known instrument, describe verbatim how it was measured. Write verbatim any *ad hoc* questions used.**  ^D14^ |
| --- | --- | --- | --- | --- |
|  |  |  |  |  |
|  |  |  |  |  |
|  |  |  |  |  |
|  |  |  |  |  |
|  |  |  |  |  |
|  |  |  |  |  |
|  |  |  |  |  |

**Part F: Results (with integrated Part E: Comparison)**

**Results for dichotomous outcomes** [e.g. death, reoperation, or any other events that either occurred or not] These outcomes are expressed as number of events per group. Print and attach additional sheets of paper if there are more outcomes, and for each new comparison of different interventions [e.g. some reviews may compare multiple interventions, such as one antibiotics vs another antibiotic as well as “any antibiotic vs any surgery”]. If an outcome is measured at several time-points enter each separately in a new row. If there are multiple result pages please enter: **Page ........ of ........**

| **Intervention (intervention 1):**  ^E1^ | | | | | | | | **Comparison intervention (Intervention 2):**  ^E2^ | | | | | | | |
| --- | --- | --- | --- | --- | --- | --- | --- | --- | --- | --- | --- | --- | --- | --- | --- |
| **Abbreviations**: OR odds ratio, RR risk ratio/relative risk, RD risk difference, HR hazard ratio, CI confidence interval, RCT randomised controlled trial, NRS non-randomised (observational) study. If more than one heterogeneity statistic is available record only one of them in this order of preference: I^2^, tau ^2^, Q, others. Record multi-arm trials in the same way as the review authors did for their meta-analysis.  **Legend**: *1= RCTs only, 2=Comparative NRS only, 3=1+2 pooled, 4=Non-comparative studies. **Across all studies pooled together. † If an outcome is measured at several time-points enter each separately in a new row. **††**If the effect estimate is >1 does this mean the intervention or the comparison was a better treatment? [e.g. Does the OR or RR of 1.25 mean that intervention is better, or the comparison is better? This should be clear either from the forest plot or how the result is described in the result section.] | | | | | | | | | | | | | | | |
| **Outco-me**  **No.**  ^D3^ | **Study types pooled**  ^F1^ [enter relevant code *] | **Number of Included studies**  ^F2^ | **Total Number of patients **** F3 | **Time point outcome measured †** [e.g. 6 months]  ^F9^ | **Meta-Analysis method** [fixed/ random]  ^F10^ | **Type of pooled effect measure** [OR, RR, RD, HR]  ^F11^ | **Larger effect size favours**  Intervention or Comparison? F4 (see notes) **††** | | **Pooled estimate **** [enter number only]  ^F12^ | **Lower end of the CI** [enter numeric value] ^F13^ | **Upper end of the CI** [enter numeric value] ^F14^ | **Heterogeneity statistics** | | | **Location of these results in paper** [e.g. T1, Fig 2, p312] |
|  |  |  |  |  |  |  |  |  |  |  |  | Statistic used [I^2^, tau ^2^, Q]  ^F15^ | Enter numerical value  ^F16^ | p-value  ^F17^ |  |
|  |  |  |  |  |  |  |  | |  |  |  |  |  |  |  |
|  |  |  |  |  |  |  |  | |  |  |  |  |  |  |  |
|  |  |  |  |  |  |  |  | |  |  |  |  |  |  |  |
|  |  |  |  |  |  |  |  | |  |  |  |  |  |  |  |
|  |  |  |  |  |  |  |  | |  |  |  |  |  |  |  |
|  |  |  |  |  |  |  |  | |  |  |  |  |  |  |  |
|  |  |  |  |  |  |  |  | |  |  |  |  |  |  |  |

**Results for continuous outcomes** [e.g. length of hospital stay, duration of operation, pain] These outcomes are usually expressed with a mean score per group, or mean difference between groups. Print and attach additional sheets of paper if there are more outcomes, and for each new comparison of different interventions. If an outcome is measured at several time-points enter each separately in a new row. If there are multiple result pages please enter: **Page ........ of ........**

| **Intervention (Intervention 1):**  ^E1^ | | | | | | | | **Comparison intervention (Intervention 2):**  ^E2^ | | | | | | | |
| --- | --- | --- | --- | --- | --- | --- | --- | --- | --- | --- | --- | --- | --- | --- | --- |
| **Abbreviations**: SD standard deviation, MD mean difference, SMD standardised mean difference, MR mean ratio/ratio of means (rarely used), WMD weighted mean difference, CI confidence interval, NRS non-randomised (observational) study. If more than one heterogeneity statistic is available record only one of them in this order of preference: I^2^, tau ^2^, Q, others. Record multi-arm trials in the same way as the review authors did for their meta-analysis.  **Legend**: *1= RCTs only, 2=Comparative NRS only, 3=1+2 pooled, 4=Non-comparative studies. **Across all studies pooled together. † If an outcome is measured at several time-points enter each separately in a new row. ††Does the effect estimate larger than 0 favour the intervention or the comparison? [e.g. Does the mean difference of + 2 mean that intervention is better, or the comparison is better? This should be clear either from the forest plot or how the result is described in the result section.] | | | | | | | | | | | | | | | |
| **Out-come**  **No.**  ^D3^ | **Study types pooled** [enter relevant code *]  ^F1^ | **Number of studies**  ^F2^ | **Total Number of patients **** F3 | **Time point outcome measured †**  ^F9^ | **Meta-analysis method** [fixed/ random]  ^F10^ | **Type of pooled effect measure** [MD,SMD, WMD, MR]  ^F11^ | **Larger effect size favours**  Intervention or Comparison? F4 †† | | **Pooled estimate **** [enter number only]  ^F12^ | **Lower CI** [enter value]  ^F13^ | **Upper CI** [enter value]  ^F14^ | **Heterogeneity statistics** | | | **Location of these results in paper** [e.g.T1, Fig1, p112] |
|  |  |  |  |  |  |  |  |  |  |  |  | Statistic used [I^2^ / tau ^2^ /Q etc.]  ^F15^ | Enter numerical value  ^F16^ | p-value  ^F17^ |  |
|  |  |  |  |  |  |  |  | |  |  |  |  |  |  |  |
|  |  |  |  |  |  |  |  | |  |  |  |  |  |  |  |
|  |  |  |  |  |  |  |  | |  |  |  |  |  |  |  |
|  |  |  |  |  |  |  |  | |  |  |  |  |  |  |  |
|  |  |  |  |  |  |  |  | |  |  |  |  |  |  |  |
|  |  |  |  |  |  |  |  | |  |  |  |  |  |  |  |
|  |  |  |  |  |  |  |  | |  |  |  |  |  |  |  |
|  |  |  |  |  |  |  |  | |  |  |  |  |  |  |  |

**Part G: Critical appraisal**

| ***Modified AMSTAR Checklist*** |  |
| --- | --- |
| **G1 Was an 'a priori' design provided?** The research question and inclusion criteria should be established before the conduct of the review.  *Note: It may be difficult to judge this without referring to a protocol, ethics approval, or pre-determined/a priori published research objectives to score a “yes.” However, look for clues in the text that state they used pre-determined criteria, predefined data-extraction form or similar. It’s fine to tick “can’t answer” if you can’t tell.* | □ Yes □ No □ Can't answer □ Not applicable |
|  | |
| **G2 Was there duplicate study selection and data extraction?** There should be at least two independent data extractors and a consensus procedure for disagreements should be in place.  *Note: 2 people do study selection, 2 people do data extraction, consensus process or one person checks the other’s work.* | □ Yes □ No □ Can't answer □ Not applicable |
|  | |
| **G3 Was a comprehensive literature search performed? *** At least two electronic sources should be searched. The report must include years and databases used (e.g., Central, EMBASE, and MEDLINE). Key words and/or MESH terms must be stated and where feasible the search strategy should be provided. All searches should be supplemented by consulting current contents, reviews, textbooks, specialized registers, or experts in the particular field of study, and by reviewing the references in the studies found.  *Note: If at least 2 sources + one supplementary strategy used, select “yes” (Cochrane register/Central counts as 2 sources; a grey literature search counts as supplementary).* | □ Yes □ No □ Can't answer □ Not applicable |
|  | |
| **G4 Was the status of publication (i.e. grey literature) used as an inclusion criterion?** The authors should state that they searched for reports regardless of their publication type. The authors should state whether or not they excluded any reports (from the systematic review), based on their publication status, language etc.  *Note: If review indicates that there was a search for “grey literature” or “unpublished literature,” indicate “yes.” SIGLE database, dissertations, conference proceedings, and trial registries are all considered grey for this purpose. If searching a source that contains both grey and non-grey, must specify that they were searching for grey/unpublished lit.* | □ Yes □ No □ Can't answer □ Not applicable |
|  | |
|  | |
| **G5 Was a list of studies (included and excluded) provided?** A list of included and excluded studies should be provided.  *Note: Acceptable if the excluded studies are referenced. If there is an electronic link to the list but the link is dead, select “no.”* | □ Yes □ No □ Can't answer □ Not applicable |
|  | |
| **G6 Were the characteristics of the included studies provided?** In an aggregated form such as a table, data from the original studies should be provided on the participants, interventions and outcomes. The ranges of characteristics in all the studies analyzed e.g., age, race, sex, relevant socioeconomic data, disease status, duration, severity, or other diseases should be reported.  *Note: Acceptable if not in table format as long as they are described as above.* | □ Yes □ No □ Can't answer □ Not applicable |
|  | |
| **G7 Was the scientific quality of the included studies assessed and documented? *** 'A priori' methods of assessment should be provided (e.g., for effectiveness studies if the author(s) chose to include only randomized, double-blind, placebo controlled studies, or allocation concealment as inclusion criteria); for other types of studies alternative items will be relevant.  *Note: Can include use of a quality scoring tool or checklist, e.g., Jadad scale, risk of bias, sensitivity analysis, etc., or a description of quality items, with some kind of result for EACH study (“low” or “high” is fine, as long as it is clear which studies scored “low” and which scored “high”; a summary score/range for all studies is not acceptable).* | □ Yes □ No □ Can't answer □ Not applicable |
|  | |
| **G8 Was the scientific quality of the included studies used appropriately in formulating conclusions? *** The results of the methodological rigor and scientific quality should be considered in the analysis and the conclusions of the review, and explicitly stated in formulating recommendations.  *Note: Might say something such as “the results should be interpreted with caution due to poor quality of included studies.” Cannot score “yes” for this question if scored “no” for question 7.* | □ Yes □ No □ Can't answer □ Not applicable |
|  | |
| **G9 Were the methods used to combine the findings of studies appropriate? *** For the pooled results, a test should be done to ensure the studies were combinable, to assess their homogeneity (i.e., Chi-squared test for homogeneity, I2). If heterogeneity exists a random effects model should be used and/or the clinical appropriateness of combining should be taken into consideration (i.e., is it sensible to combine?).  *Note: Indicate “yes” if they mention or describe heterogeneity, i.e., if they explain that they cannot pool because of heterogeneity/variability between interventions.* | □ Yes □ No □ Can't answer □ Not applicable |
|  | |
| **G10 Was the likelihood of publication bias assessed?** An assessment of publication bias should include a combination of graphical aids (e.g., funnel plot, other available tests) and/or statistical tests (e.g., Egger regression test, Hedges-Olken).  *Note: If no test values or funnel plot included, score “no”. Score “yes” if mentions that publication bias could not be assessed because there were fewer than 10 included studies.* | □ Yes □ No □ Can't answer □ Not applicable |
|  | |
| **G11 Was the conflict of interest included?** Potential sources of support should be clearly acknowledged in both the systematic review and the included studies.  *Note: To get a “yes,” must indicate source of funding or support for the systematic review AND for each of the included studies.* | □ Yes □ No □ Can't answer □ Not applicable |
|  | |
| Adapted from Shea *et al.* *BMC Medical Research Methodology* 2007 **7**:10   doi:10.1186/1471-2288-7-10 | |

**G12 Are the conclusions of the review consistent with the presented results?** [Additional non-AMSTAR item]

Yes □ No □ Unsure/Unclear/Can’t answer □

^6.12a^ Explanation/comment (if needed):...............................................................................................................

.............................................................................................................................................................................

**G13 Overall risk of bias judgment for the review ***

You need to have answered ‘yes’ or ‘not applicable’ to all 4 AMSTAR domains above marked with an asterisk *(items 3, 7, 8, and 9) to pass a ‘low risk of bias’ judgement. A single ‘No’ answer is sufficient to judge High risk. Any ‘Can’t answer’ answers would yield the unclear risk judgment, unless the answer to any of the questions is No (high risk).

**Low risk Unclear risk High risk**

**A14 Data extractor’s comments** [about anything in the form]

……………………………………………………………......................................................................................................

……………………………………………………………......................................................................................................

.....................................................................................................................................................................

.....................................................................................................................................................................

.....................................................................................................................................................................

**Additional potentially eligible papers identified in this paper**

Please read the Introduction and Discussion sections carefully to identify additional papers that could be eligible for inclusion in our review. Authors will usually refer to previous systematic reviews of the same condition / interventions /diagnostic tests / economic evaluations and these may qualify for screening for our review. **Please circle such potentially relevant references in the References section of the paper you are extracting and hand the annotated paper back with this form.**

**Appendix S4** List of excluded studies with reasons

**Unobtainable reports**

Abdulwadud O. *Proton Pump Inhibitors* versus *H2 Antagonists or Placebo in Treating Bleeding Peptic Ulcer.* Centre for Clinical Effectiveness (CCE): Clayton; 11, 2002.

Conseil d'Evaluation des Technologies de la Sante du Québec. *The Costs of Conventional Cholecystectomy, Laparoscopic Cholecystectomy and Biliary Lithotripsy – Nonsystematic Review*. Conseil d'Evaluation des Technologies de la Sante du Québec: Montreal, 1993.

HAYES, Inc. *Capsule endoscopy of the small bowel for obscure gastrointestinal bleeding*. HAYES: Lansdale, 2013.

HAYES, Inc. *Endoloop ligature (Ethicon Endo-Surgery Inc.) for appendiceal stump closure during laparoscopic appendectomy.* HAYES: Lansdale, 2013.

Muggli E. *Antibiotic Therapy for Acute Appendicitis: Ampicillin, Metronidazole plus Gentamycin* versus *Cephalosporin*. Centre for Clinical Effectiveness (CCE): Clayton: 12; 2002.

Netherlands Organisation for Health Research and Development (ZonMw). *Identifying Optimal Diagnostic Strategies Algorithms with Multiple Tests. Ancillary Study of Optimisation of Diagnostic Imaging Use in Patients with Acute Abdominal Pain: Cost-Effectiveness Analysis of Imaging Strategies (OPTIMA Study).* ZonMw: The Hague, 2000.

Netherlands Organisation for Health Research and Development (ZonMw). *Optimization of Diagnostic Imaging Use in Patients with Acute Abdominal Pain: Cost-Effectiveness Analysis of Imaging Strategies (OPTIMA Study)*. ZonMw: The Hague, 2000.

**Non-English language papers**

Ambrosi A, Fabiano G, Sparasci V, Morelli M, Pezzolla A, Errico D *et al.* [Comparison of hernioplasty using the technique of Bassini and Trabucco: comparative analysis and results.] *Ann Ital Chir* 1998; **69**: 203–206.

Angelescu N, Jitea N, Burcos T, Cristian D, Voiculescu S, Dimitriu C. [A comparative study of the laparoscopic and classic treatments of inguinal hernias.] *Chirurgia* 1996; **45**: 267–270.

Angenete E, Skullman S, Jivegard L, Sjovall H, Alopaeus E, Svanberg T. *Laparoskopiskt lavage av patienter med perforerad divertikulit.* [*Laparoscopic peritoneal lavage for patients with perforated diverticulitis.*] HTA-rapport 2010:25. Regional Health Technology Assessment Centre (HTA-centrum): Gothenburg, 2010.

Basurto OX, Robles PL. Anti-inflammatory drugs for biliary colics: systematic review and meta-analysis of randomized controlled trials. *Gastroenterol Hepatol* 2008; **31**: 1–7.

Chai C, Cao N, Li Q, Yang KH. Endoscopic treatment of acute biliary pancreatitis: a systematic review. *World Chin J Digestol* 2010; **18**: 404–408.

Chen SM, Xiong GS, Wu SM. [A meta-analysis on the timing of parenteral nutrition and enteral nutrition in acute pancreatitis.] *Chin J Clin Nutr* 2012; **20**: 363–368.

Chen Y, Qu L, Li X, Feng W. [Meta analysis of efficacy of omeprazole *versus* famotidine in treatment of duodenal ulcer.] *Pharma Care Res* 2012; **12**: 67–69.

Cheng XZ, Guo TK, Da MX, Jing WT, Hu DP. [Stilamin for intestinal obstruction: a systematic review of efficacy.] *World Chin J Digestol* 2012; **20**: 2511–2518.

Danish Centre for Evaluation and Health Technology Assessment. Hospital conference from an HTA perspective: acute appendicitis in adults (funded by DIHTA), 1998.

Del Cura JL, Oleaga L, Grande D, Vela AC, Ibanez AM. Reliability of diagnostic imaging techniques in suspected acute appendicitis: proposed diagnostic protocol. *Radiologia* 2001; **43**: 478–489.

Feng SF, Tang SH, Zhang XJ. Tolerance and efficacy of nasogastric enteral nutrition for severe acute pancreatitis: a meta-analysis. *Med J Chin PLA* 2013; **38**: 141–146.

Ferrante D. *Laparoscopic* Vs *Conventional Appendectomy for Suspected Acute Appendicitis*. Informe de Respuesta Rapida No. 2. Institute for Clinical Effectiveness and Health Policy (IECS): Buenos Aires, 2003.

Ferrante D. *Conventional* Vs *Laparoscopic Hernioplasty for the Treatment of Inguinal Hernias*. . Informe de Respuesta Rapida No.3. Institute for Clinical Effectiveness and Health Policy (IECS): Buenos Aires, 2004.

Ge W, Ma B, Yang KH, Zhao FH, Zhang J, Tian JH. Kansui root for treating severe acute pancreatitis: a systematic review. *Chin J Evid Based Med* 2009; **9**: 964–968.

Gerhardus A, Jalilvand N, Heintze C, Krauth C. *The Open* Versus *Laparoscopic Methods in Surgery of Inguinal Hernias – A Systematic Review.* Volume 30. Hannover Medical School, Medizinische Hochschule Hannover (MHH): Hannover**,** 2003.

Guo D, Jia C, Xu ZH, Zhu LG. Effectiveness of enteral immunonutrition in acute pancreatitis: a systematic review. *Chin J Evid Based Med* 2013; **13**: 346–351.

Hay JM. [Symptomatic common bile duct lithiasis: endoscopy or open surgery?] *J Chir* 1998; **135**: 4–9.

Healthcare Insurance Board. Laparoscopic surgery for inguinal hernia *versus* conventional operative treatment – primary research, 1998.

Izbicki JR, Gawad KA, Quirrenbach S, Hosch SB, Breid V, Knoefel WT *et al.* [Can stapled anastomosis in visceral surgery still be justified: a prospective controlled randomized study of the cost-effectiveness of hand-sewn and stapled anastomoses.] *Chirurg* 1998; **69**: 725–734.

Jiang K, Chen XZ, Xia Q, Tang WF, Wang L. Early veno-venous hemofiltration for severe acute pancreatitis: a systematic review. *Chin J Evid Based Med* 2007; **7**: 121–134.

Jing EY, Liu YL, Yang KH, Guo TK. Laparoscopic compared with open methods of groin hernia repair in adults: a systematic review of clinical controlled trials. *Chin J Evid Based Med* 2010; **10**: 875–881.

Kuwabara K, Imanaka Y, Matsuda S, Fushimi K, Hashimoto H, Ishikawa KB *et al.* Cost of open *versus* laparoscopic appendectomy. *Clin Ter* 2008; **159**: 155–163.

L'Agence Nationale d'Accreditation d'Evaluation en Sante (ANAES). *Clinical and Economic Evaluation of Laparoscopic Surgery in the Context of Inguinal Hernia Repair.* ANAES: Saint-Denis La Plaine, 2000.

Li BH, Wang WH, Zhou HQ. Comparison on continuous regional arterial infusion and intravenous administration in the treatment of severe acute pancreatitis: a systematic review. *Chin J Interv Imaging Ther* 2013; **10**: 19–23.

Li ZY, Wu B, Zhang L, Wu HM. Evidence-based diagnosis of small bowel obstruction with computed tomography. *Chin J Evid Based Med* 2007; **7**: 311–315.

Miao B, Cui NQ, Li ZL, Ma T, Zhao G, Wang X. Systematic evaluation of the therapeutic efficacy of Tongli Gongxia herbs on severe acute pancreatitis. *World Chin J Digestol* 2009; **17**: 1042–1047.

Minutolo V, Gagliano G, Minutolo O, Carnazza M, La TS, Buttafuoco A *et al.* Laparoscopic appendectomy for acute appendicitis. *Chir Ital* 2009; **61**: 591–596.

Pichon RA, Augustovski F, Bardach A, Garcia MS, Lopez A, Glujovsky D. Laparoscopy usefulness in the management of biliary tract stones, 2005.

Rao CY, Hu CL, Zhao XY. [Role of prophylactic antibiotics in the management of acute necrotizing pancreatitis: a meta-analysis.] *World Chin J Digestol* 2012; **20**: 1246–1251.

Salm R, Grund KE, Szucs TD. [Endoscopic hemostasis with fibrin glue in peptic ulcer hemorrhage: economic aspects.] *Zentralbl Chir* 1996; **121**: 847–850.

Saviano MS, Piccoli M, Heydari A, Gelmini R, Pezcoller C, Guarasci N. [Costs of cholecystectomy after the arrival of video-laparoscopic surgery.] *Ann Ital Chir* 1996; **67**: 463–468.

Sheng YY, Zou XP, Yu CG, Lv Y, Zhang LL. Adjuvant treatment of severe acute pancreatitis with rhubarb: a systematic review. *World Chin J Digestol* 2010; **18**: 730–735.

Vauth C, Englert H, Fischer T, Kulp W, Greiner W, Willich SN *et al.* Sonographische Diagnostik beim akuten Abdomen bei Kindern und Erwachsenen [Sonographic diagnosis of acute abdomen in children and adults]. *GMS Health Technol Assess* 2005; **1**: Doc08. <http://www.egms.de/en/journals/hta/2005-1/hta000008.shtml> [accesed 27 January 2017].

Wang XJ, Zeng XT, Tian Y, Ni SZ, Xiao M. [Effectiveness and safety of octreotide combined with ulinastatin for treating acute pancreatitis in China: a meta-analysis]. *Chin J Evid Based Med* 2011; **11**: 1302–1312.

Wang YP, Li DB, Dong CL, Wu XA. Antibiotic prophylaxis in severe acute pancreatitis: a systematic review. *Chin J Evid Based Med* 2012; **12**: 477–483.

Xiao QC, Wu TX, Dui DH, Lan TG, Li JH. Fat emulsion for acute pancreatitis: a systematic review. *Chin J Evid Based Med* 2009; **9**: 452–457.

Yan ZF, Qi YM, Lu W, Xie Y. Nasogastric enteral nutrition for severe acute pancreatitis: meta-analysis of clinical trials. *Chin J Clin Nutr* 2009; **17**: 271–274.

Yang JL, Guo Z, Wu ZY, Wang YP, Zeng C. Ulinastatin for acute pancreatitis: a systematic review. *Chin J Evid Based Med* 2005; **5**: 323–330.

Yin J, Zhou Q, Ling Y. Calculus removed for common bile duct stones: a meta-analysis. *J Xi'an Jiaotong Univ (Med Sci)* 2008; **29**: 90–93.

Yuan S, Sun DY. Application of bedside emergency ERCP in the treatment of severe acute biliary pancreatitis. *World Chin J Digestol* 2013; **21**: 2217–2220.

Zhang RX, Zheng Z, He T, Wang Y, Qu BH, Zheng XL *et al.* Primary suture *versus* T-tube drainage after laparoscopic common bile duct stone exploration: a systematic review. *Chin J Evid Based Med* 2011; **11**: 1161–1165.

Zhao ZF, Gao HL, Yao P. Ursodeoxycholic acid for treatment of bile reflux gastritis: a systematic review. *World Chin J Digestol* 2013; **21**: 2708–2716.

Zhou J, He JD, Ouyang XB, Wang YP. Erythromycin infusion prior to emergency endoscopy for acute upper gastrointestinal bleeding: a systematic review. *World Chin J Digestol* 2009; **17**: 3273–3277.

Zhou JP, Liu Q. Tension-free hernioplasty for groin hernia in adult: a meta-analysis. *Chin J Evid Based Med* 2005; **5**: 303–310.

Zuo YY, Kang Y. [Total enteral nutrition *versus* total parenteral nutrition for patients with severe acute pancreatitis: a meta-analysis.] *Chin J Evid Based Med* 2011; **11**: 1295–1301.

**Studies included in overviews of diagnostic studies**

Al-Khayal KA, Al-Omran MA. Computed tomography and ultrasonography in the diagnosis of equivocal acute appendicitis. a meta-analysis. *Saudi Med J* 2007; **28**: 173–180.

Andersson RE. Meta-analysis of the clinical and laboratory diagnosis of appendicitis. *Br J Surg* 2004; **91**: 28–37.

Barger RL, Nandalur KR. Diagnostic performance of magnetic resonance imaging in the detection of appendicitis in adults: a meta-analysis. *Acad Radiol* 2010; **17**: 1211–1216.

Basaran A, Basaran M. Diagnosis of acute appendicitis during pregnancy: a systematic review. *Obstet Gynecol Surv* 2009; **64**: 481–488.

Burcharth J, Pommergaard HC, Rosenberg J, Gögenur I. Hyperbilirubinemia as a predictor for appendiceal perforation: a systematic review. *Scand J Surg* 2013; **102**: 55–60.

Carroll PJ, Gibson D, El-Faedy O, Dunne C, Coffey C, Hannigan A *et al.* Surgeon-performed ultrasound at the bedside for the detection of appendicitis and gallstones: systematic review and meta-analysis. *Am J Surg* 2013; **205**: 102–108.

Chang K, Lu W, Zhang K, Jia S, Li F, Wang F *et al.* Rapid urinary trypsinogen-2 test in the early diagnosis of acute pancreatitis: a meta-analysis. *Clin Biochem* 2012; **45**: 1051–1056.

Chua AE, Ridley LJ. Diagnostic accuracy of CT angiography in acute gastrointestinal bleeding. *J Med Imaging Radiat Oncol* 2008; **52**: 333–338.

Cobben L, Groot I, Kingma L, Coerkamp E, Puylaert J, Blickman J. A simple MRI protocol in patients with clinically suspected appendicitis: results in 138 patients and effect on outcome of appendectomy. *Eur Radiol* 2009. **19**: 1175–1183.

Currie GM. Cost-effectiveness analysis of subtraction scintigraphy in patients with acute lower gastrointestinal tract hemorrhage. *J Nucl Med Technol* 2007; **35**: 140–147.

De LS, Leandro G, Buscarini E. Endoscopic ultrasonography *versus* endoscopic retrograde cholangiopancreatography in acute biliary pancreatitis: a systematic review. *Eur J Gastroenterol Hepatol* 2011; **23**: 367–374.

Doria AS, Moineddin R, Kellenberger CJ, Epelman M, Beyene J, Schuh S *et al.* US or CT for diagnosis of appendicitis in children and adults: a meta-analysis. *Radiology* 2006; **241**: 83–94.

Evennett NJ, Petrov MS, Mittal A, Windsor JA. Systematic review and pooled estimates for the diagnostic accuracy of serological markers for intestinal ischemia. *World J Surg* 2009; **33**: 1374–1383.

Fujii Y, Hata J, Futagami K, Hamada T, Mitsuoka H, Teramen K *et al.* Ultrasonography improves diagnostic accuracy of acute appendicitis and provides cost savings to hospitals in Japan. *J Ultrasound Med* 2000; **19**: 409–414.

García-Blázquez V, Vicente-Bártulos A, Olavarria-Delgado A, Plana MN, Van Der Winden D, Zamora J. Accuracy of CT angiography in the diagnosis of acute gastrointestinal bleeding: systematic review and meta-analysis. *Eur Radiol* 2013; **23**: 1181–1190.

Giordano S, Pääkkönen M, Salminen P, Grönroos JM. Elevated serum bilirubin in assessing the likelihood of perforation in acute appendicitis: a diagnostic meta-analysis. *Int J Surg* 2013; **11**: 795–800.

Hallan S, Asberg A. The accuracy of C-reactive protein in diagnosing acute appendicitis. *Scand J Clin Lab Invest* 1997; **57**: 373–380.

Hlibczuk V, Dattaro JA, Jin Z, Falzon L, Brown MD. Diagnostic accuracy of noncontrast computed tomography for appendicitis in adults: a systematic review. *Ann Emerg Med* 2010; **55**: 51–59.

Jin T, Huang W, Jiang K, Xiong JJ, Xue P, Javed MA *et al.* Urinary trypsinogen-2 for diagnosing acute pancreatitis: a meta-analysis. *Hepatobiliary Pancreatic Dis Int* 2013; **12**: 355–362.

Kiewiet JJ, Leeuwenburgh MM, Bipat S, Bossuyt PM, Stoker J, Boermeester MA. A systematic review and meta-analysis of diagnostic performance of imaging in acute cholecystitis. *Radiology* 2012; **264**: 708–720.

Krajewski S, Brown J, Phang PT, Raval M, Brown CJ. Impact of computed tomography of the abdomen on clinical outcomes in patients with acute right lower quadrant pain: a meta-analysis. *Can J Surg* 2011; **54**: 43–53.

Laméris W, van Randen A, Bipat S, Bossuyt PM, Boermeester MA, Stoker J. Graded compression ultrasonography and computed tomography in acute colonic diverticulitis: meta-analysis of test accuracy. *Eur Radiol* 2008; **18**: 2498–2511.

Liljegren G, Chabok A, Wickbom M, Smedh K, Nilsson K. Acute colonic diverticulitis: a systematic review of diagnostic accuracy. *Colorectal Dis* 2007; **9**: 480–488.

Mallo RD, Salem L, Lalani T, Flum DR. Computed tomography diagnosis of ischemia and complete obstruction in small bowel obstruction: a systematic review. *J Gastrointest Surg* 2005; **9**: 690–694.

Neumayer L, Kennedy A. Imaging in appendicitis: a review with special emphasis on the treatment of women. *Obstet Gynecol* 2003; **102**: 1404–1409.

Ohle R, O’Reilly F, O’Brien KK, Fahey T, Dimitrov BD. The Alvarado score for predicting acute appendicitis: a systematic review. *BMC Med* 2011; **9**: 139.

Orr RK, Porter D, Hartman D. Ultrasonography to evaluate adults for appendicitis: decision making based on meta-analysis and probabilistic reasoning. *Acad Emerg Med* 1995; **2**: 644–650.

Petrov MS, Savides TJ. Systematic review of endoscopic ultrasonography *versus* endoscopic retrograde cholangiopancreatography for suspected choledocholithiasis. *Br J Surg* 2009; **96**: 967–974.

Rao PM, Rhea JT, Novelline RA, Mostafavi AA, McCabe CJ. Effect of computed tomography of the appendix on treatment of patients and use of hospital resources. *N Engl J Med* 1998; **338**: 141–146.

Romagnuolo J, Bardou M, Rahme E, Joseph L, Reinhold C, Barkun AN. Magnetic resonance cholangiopancreatography: a meta-analysis of test performance in suspected biliary disease. *Ann Intern Med* 2003; **139**: 547–557.

Ross M, Brown M, McLaughlin K, Atkinson P, Thompson J, Powelson S *et al.* Emergency physician-performed ultrasound to diagnose cholelithiasis: a systematic review. *Acad Emerg Med* 2011; **18**: 227–235.

Taylor MR, Lalani N. Adult small bowel obstruction. *Acad Emerg Med* 2013; **20**: 528–544.

Terasawa T, Blackmore C, Bent S, Kohlwes RJ. Systematic review: computed tomography and ultrasonography to detect acute appendicitis in adults and adolescents. *Ann Intern Med* 2004; **141**: 537–546.

Trowbridge RL, Rutkowski NK, Shojania KG. Does this patient have acute cholecystitis? *JAMA* 2003; **289**: 80–86.

Tse F, Liu L, Barkun AN, Armstrong D, Moayyedi P. EUS: a meta-analysis of test performance in suspected choledocholithiasis. *Gastrointest Endosc* 2008; **67**: 235–244.

van Randen A, Bipat S, Zwinderman AH, Ubbink DT, Stoker J, Boermeester MA. Acute appendicitis: meta-analysis of diagnostic performance of CT and graded compression US related to prevalence of disease. *Radiology* 2008; **249**: 97–106.

Verma D, Kapadia A, Eisen GM, Adler DG. EUS *vs* MRCP for detection of choledocholithiasis. *Gastrointest Endosc* 2006; **64**: 248–254.

Wang Z, Chen JQ, Liu JL, Qin XG, Huang Y. CT enterography in obscure gastrointestinal bleeding: a systematic review and meta-analysis. *J Med Imaging Radiat Oncol* 2013; **57**: 263–273.

Weston AR, Jackson TJ, Blamey S. Diagnosis of appendicitis in adults by ultrasonography or computed tomography: a systematic review and meta-analysis. *Int J Technol Assess Health Care* 2005; **21**: 368–379.

Yu CW, Juan LI, Wu MH, Shen CJ, Wu JY, Lee CC. Systematic review and meta-analysis of the diagnostic accuracy of procalcitonin, C-reactive protein and white blood cell count for suspected acute appendicitis. *Br J Surg* 2013; **100**: 322–329.

Yu SH, Kim CB, Park JW, Kim MS, Radosevich DM. Ultrasonography in the diagnosis of appendicitis: evaluation by meta-analysis. *Korean J Radiol* 2005; **6**: 267–277.

**Studies included in overviews of economic evaluations**

Al-Sabah S, Barkun AN, Herba K, Adam V, Fallone C, Mayrand S *et al.* Cost-effectiveness of proton-pump inhibition before endoscopy in upper gastrointestinal bleeding. *Clin Gastroenterol Hepatol* 2008; **6**: 418–425.

Ali R, Khan MR, Pishori T, Tayeb M. Laparoscopic appendectomy for acute appendicitis: is this a feasible option for developing countries. *Saudi J Gastroenterol* 2010; **16**: 25–29.

Arslan K, Eryilmaz MA, Okuş A, Doğru O, Karahan O, Köksal H. Is total–subtotal colectomy and primary anastomosis a good treatment alternative in malignant obstructive lesions of the left colon? *Turk J Trauma Emerg Surg* 2012; **18**: 311–316.

Artifon EL, da Silveira EB, Aparicio D, Takada J, Baracat R, Sakai CM *et al.* Management of common bile duct stones in cirrhotic patients with coagulopathy: a comparison of supra-papillary puncture and standard cannulation technique. *Dig Dis Sci* 2011; **56**: 1904–1911.

Atreja A, Fu AZ, Sanaka MR, Vargo JJ. Non-invasive testing for *Helicobacter pylori* in patients hospitalized with peptic ulcer hemorrhage: a cost-effectiveness analysis. *Dig Dis Sci* 2010; **55**: 1356–1363.

Baik SM, Hong KS, Kim YI. A comparison of transumbilical single-port laparoscopic appendectomy and conventional three-port laparoscopic appendectomy: from the diagnosis to the hospital cost. *J Korean Surg Soc* 2013; **85**: 68–74.

Barkun AN, Adam V, Sung JJ, Kuipers EJ, Mössner J, Jensen D *et al.* Cost effectiveness of high-dose intravenous esomeprazole for peptic ulcer bleeding. *Pharmacoeconomics* 2010; **28**: 217–230.

Barkun AN, Herba K, Adam V, Kennedy W, Fallone CA, Bardou M. The cost-effectiveness of high-dose oral proton pump inhibition after endoscopy in the acute treatment of peptic ulcer bleeding. *Aliment Pharmacol Ther* 2004; **20**: 195–202.

Barkun AN, Herba K, Adam V, Kennedy W, Fallone CA, Bardou M. High-dose intravenous proton pump inhibition following endoscopic therapy in the acute management of patients with bleeding peptic ulcers in the USA and Canada: a cost-effectiveness analysis. *Aliment Pharmacol Ther* 2004; **19**: 591–600.

Brasel KJ, Borgstrom DC, Weigelt JA. Cost–utility analysis of contaminated appendectomy wounds. *J Am Coll Surg* 1997; **184**: 23–30.

Brasel KJ, Borgstrom DC, Weigelt JA. Management of penetrating colon trauma: a cost-utility analysis. *Surgery* 1999; **125**: 471–479.

Bresciani C, Perez RO, Habr-Gama A, Jacob CE, Ozaki A, Batagello C *et al.* Laparoscopic *versus* standard appendectomy outcomes and cost comparisons in the private sector. *J Gastrointest Surg* 2005; **9**: 1174–1181.

Chen BH, Liu HW, Huang SL, Lin FC, Dai MG, Chen YL *et al.* Comparison of appendectomy medical expense and clinical outcome between fee for service and prospective payment system. *Kaohsiung J Med Sci* 2000; **16**: 293–298.

Chu DI, Gainsbury ML, Howard LA, Stucchi AF, Becker JM. Early *versus* late adhesiolysis for adhesive-related intestinal obstruction: a nationwide analysis of inpatient outcomes. *J Gastrointest Surg* 2013; **17**: 288–297.

Costa-Navarro D, Jiménez-Fuertes M, Illán-Riquelme A. Laparoscopic appendectomy: quality care and cost-effectiveness for today’s economy. *World J Emerg Surg* 2013; **8**: 45.

Delibegović S. The use of a single hem-o-lok clip in securing the base of the appendix during laparoscopic appendectomy. *J Laparoendosc Adv Surg Tech A* 2012; **22**: 85–87.

Enns RA, Gagnon YM, Rioux KP, Levy AR. Cost-effectiveness in Canada of intravenous proton pump inhibitors for all patients presenting with acute upper gastrointestinal bleeding. *Aliment Pharmacol Ther* 2003; **17**: 225–233.

Erstad BL. Cost-effectiveness of proton pump inhibitor therapy for acute peptic ulcer-related bleeding. *Crit Care Med* 2004; **32**: 1277–1283.

Ge B, Zhao H, Chen Q, Jin W, Liu L, Huang Q. A randomized comparison of gasless laparoscopic appendectomy and conventional laparoscopic appendectomy. *World J Emerg Surg* 2014; **9**: 3.

Ghoshal UC, Aggarwal R, Baba CS. Recurrent duodenal ulcer haemorrhage: a pharmacoeconomic comparison of various management strategies. *Expert Opin Pharmacother* 2003; **4**: 1593–1603.

Gomez D, Addison A, De Rosa A, Brooks A, Cameron IC. Retrospective study of patients with acute pancreatitis: is serum amylase still required? *BMJ Open* 2012; **2**: e001471.

Govindarajan A, Naimark D, Coburn NG, Smith AJ, Law CH. Use of colonic stents in emergent malignant left colonic obstruction: a Markov chain Monte Carlo decision analysis. *Dis Colon Rectum* 2007; **50**: 1811–1824.

Gowen GF. Rapid resolution of small-bowel obstruction with the long tube, endoscopically advanced into the jejunum. *Am J Surg* 2007; **193**: 184–189.

Gralnek IM, Jensen DM, Kovacs TO, Jutabha R, Jensen ME, Cheng S *et al.* An economic analysis of patients with active arterial peptic ulcer hemorrhage treated with endoscopic heater probe, injection sclerosis, or surgery in a prospective, randomized trial. *Gastrointest Endosc* 1997; **46**: 105–112.

Gutt CN, Encke J, Köninger J, Harnoss JC, Weigand K, Kipfmüller K *et al.* Acute cholecystitis: early *versus* delayed cholecystectomy, a multicenter randomized trial (ACDC Study, NCT00447304). *Ann Surg* 2013; **258**: 385–391.

Haas L, Stargardt T, Schreyoegg J. Cost-effectiveness of open *versus* laparoscopic appendectomy: a multilevel approach with propensity score matching. *Eur J Health Econ* 2012; **13**: 549–560.

Harrell AG, Lincourt AE, Novitsky YW, Rosen MJ, Kuwada TS, Kercher KW *et al.* Advantages of laparoscopic appendectomy in the elderly. *Am Surg* 2006; **72**: 474–480.

Heikkinen TJ, Haukipuro K, Hulkko A. Cost-effective appendectomy: open or laparoscopic? A prospective randomized study. *Surg Endosc* 1998; **12**: 1204–1208.

Hue CS, Kim JS, Kim KH, Nam SH, Kim KW. The usefulness and safety of Hem-o-lok clips for the closure of appendicular stump during laparoscopic appendectomy. *J Korean Surg Soc* 2013; **84**: 27–32.

Hultman CS, Herbst CA, McCall JM, Mauro MA. The efficacy of percutaneous cholecystostomy in critically ill patients. *Am Surg* 1996; **62**: 263–269.

Imperiale TF, Kong N. Second-look endoscopy for bleeding peptic ulcer disease: a decision-effectiveness and cost-effectiveness analysis. *J Clin Gastroenterol* 2012; **46**: e71–e75.

Jeurnink SM, Polinder S, Steyerberg EW, Kuipers EJ, Siersema PD. Cost comparison of gastrojejunostomy *versus* duodenal stent placement for malignant gastric outlet obstruction. *J Gastroenterol* 2010; **45**: 537–543.

Jiang X, Meng HB, Zhou DL, Ding WX, Lu LS. Comparison of clinical outcomes of open, laparoscopic and single port appendicectomies. *Ann R Coll Surg Engl* 2013; **95**: 468–472.

Johansson M, Thune A, Nelvin L, Stiernstam M, Westman B, Lundell L. Randomized clinical trial of open *versus* laparoscopic cholecystectomy for acute cholecystitis. *Br J Surg* 2005; **92**: 44–49.

Johner A, Raymakers A, Wiseman SM. Cost utility of early *versus* delayed laparoscopic cholecystectomy for acute cholecystitis. *Surg Endosc Other Intervent Tech* 2013; **27**: 256–262.

Kald A, Kullman E, Anderberg B, Wirén M, Carlsson P, Ringqvist I *et al.* Cost-minimisation analysis of laparoscopic and open appendicectomy. *Eur J Surg* 1999; **165**: 579–582.

Kaplan M, Salman B, Yilmaz TU, Oguz M. A quality of life comparison of laparoscopic and open approaches in acute appendicitis: a randomised prospective study. *Acta Chir Belg* 2009; **109**: 356–363.

Kastenberg ZJ, Hurley MP, Luan A, Vasu-Devan V, Spain DA, Owens DK *et al.* Cost-effectiveness of preoperative imaging for appendicitis after indeterminate ultrasonography in the second or third trimester of pregnancy. *Obstet Gynecol* 2013; **122**: 821–829.

Kehagias I, Karamanakos SN, Panagiotopoulos S, Panagopoulos K, Kalfarentzos F. Laparoscopic *versus* open appendectomy: which way to go?" *World J Gastroenterol* 2008; **14**: 4909–4914.

Khaikin M, Schneidereit N, Cera S, Sands D, Efron J, Weiss EG *et al.* Laparoscopic *vs* open surgery for acute adhesive small-bowel obstruction: patients’ outcome and cost-effectiveness. *Surg Endosc* 2007; **21**: 742–746.

Kiudelis M, Ignatavicius P, Zviniene K, Grizas S. Analysis of intracorporeal knotting with invaginating suture *versus* endoloops in appendiceal stump closure. *Wideochir Inne Tech Maloinwazyjne* 2013; **8**: 69–73.

Kurtz RJ, Heimann TM. Comparison of open and laparoscopic treatment of acute appendicitis. *Am J Surg* 2001; **182**: 211–214.

Laine L, Shah A. Randomized trial of urgent *vs.* elective colonoscopy in patients hospitalized with lower GI bleeding. *Am J Gastroenterol* 2010; **105**: 2636–2641.

Lee JS, Hong TH. Comparison of various methods of mesoappendix dissection in laparoscopic appendectomy. *J Laparoendosc Adv Surg Tech* *A* 2014; **24**: 28–31.

Lee KK, You JH, Wong IC, Kwong SK, Lau JM, Chan TY *et al.* Cost-effectiveness analysis of high-dose omeprazole infusion as adjuvant therapy to endoscopic treatment of bleeding peptic ulcer. *Gastrointest Endosc* 2003; **57**: 160–164.

Lee YS, Kim JH, Moon EJ, Kim JJ, Lee KH, Oh SJ *et al.* Comparative study on surgical outcomes and operative costs of transumbilical single-port laparoscopic appendectomy *versus* conventional laparoscopic appendectomy in adult patients. *Surg Laparosc Endosc Percutan Tech* 2009; **19**: 493–496.

Long KH, Bannon MP, Zietlow SP, Helgeson ER, Harmsen WS, Smith CD *et al.* A prospective randomized comparison of laparoscopic appendectomy with open appendectomy: clinical and economic analyses. *Surgery* 2001; **129**: 390–400.

Lu J, Xiong XZ, Cheng Y, Lin YX, Zhou RX, You Z *et al.* One-stage *versus* two-stage management for concomitant gallbladder stones and common bile duct stones in patients with obstructive jaundice. *Am Surg* 2013; **79**: 1142–1148.

Macafee DA, Humes DJ, Bouliotis G, Beckingham IJ, Whynes DK, Lobo DN. Prospective randomized trial using cost–utility analysis of early *versus* delayed laparoscopic cholecystectomy for acute gallbladder disease. *Br J Surg* 2009; **96**: 1031–1040.

Macarulla E, Vallet J, Abad JM, Hussein H, Fernández E, Nieto B. Laparoscopic *versus* open appendectomy: a prospective randomized trial. *Surg Laparosc Endosc* 1997; **7**: 335–339.

Martin LC, Puente I, Sosa JL, Bassin A, Breslaw R, McKenney MG *et al.* Open *versus* laparoscopic appendectomy: a prospective randomised comparison. *Ann Surg* 1995; **222**: 256–262.

Masoomi H, Stamos MJ, Carmichael JC, Nguyen B, Buchberg B, Mills S. Does primary anastomosis with diversion have any advantages over Hartmann’s procedure in acute diverticulitis? *Dig Surg* 2012; **29**: 315–320.

McCahill LE, Pellegrini CA, Wiggins T, Helton WS. A clinical outcome and cost analysis of laparoscopic *versus* open appendectomy. *Am J Surg* 1996; **171**: 533–537.

McGrath B, Buckius MT, Grim R, Bell T, Ahuja V. Economics of appendicitis: cost trend analysis of laparoscopic *versus* open appendectomy from 1998 to 2008. *J Surg Res* 2011; **171**: e161–e168.

McGregor M. *Should the MUHC Approve the Use of Colorectal Stents?* Report No. 6; The Technology Assessment Unit (TAU) of the McGill University Health Centre (MUHC): Montreal; 2003. <https://www.mcgill.ca/tau/files/tau/Stents_colorectal_Feb_2003.pdf> [accessed 27 January 2017].

Merhoff AM, Merhoff GC, Franklin ME. Laparoscopic *versus* open appendectomy. *Am J Surg* 2000; **179**: 375–378.

Minné L, Varner D, Burnell A, Ratzer E, Clark J, Haun W. Laparoscopic *vs* open appendectomy: prospective randomized study of outcomes. *Arch Surg* 1997; **132**: 708–711.

Moore DE, Speroff T, Grogan E, Poulose B, Holzman MD. Cost perspectives of laparoscopic and open appendectomy. *Surg Endosc* 2005; **19**: 374–378.

Nakhamiyayev V, Galldin L, Chiarello M, Lumba A, Gorecki PJ. Laparoscopic appendectomy is the preferred approach for appendicitis: a retrospective review of two practice patterns. *Surg Endosc Other Intervent Tech* 2010; **24**: 859–864.

Nazzal M, Ali MA, Turfah F, Kaidi A, Saba A, Pleatman M *et al.* Laparoscopic appendectomy: a viable alternative approach. *J Laparoendosc Adv Surg Tech A* 1997; **7**: 1–6.

Oberkofler CE, Rickenbacher A, Raptis DA, Lehmann K, Villiger P, Buchli C *et al.* A multicenter randomized clinical trial of primary anastomosis or Hartmann’s procedure for perforated left colonic diverticulitis with purulent or fecal peritonitis. *Ann Surg* 2012; **256**: 819–827.

Oyasiji T, Angelo S, Kyriakides TC, Helton SW. Small bowel obstruction: outcome and cost implications of admitting service. *Am Surg* 2010; **76**: 687–691.

Partecke LI, Kessler W, von Bernstorff W, Diedrich S, Heidecke CD, Patrzyk M. Laparoscopic appendectomy using a single polymeric clip to close the appendicular stump. *Langenbecks Arch Chir* 2010; **395**: 1077–1082.

Richards KF, Fisher KS, Flores JH, Christensen BJ. Laparoscopic appendectomy: comparison with open appendectomy in 720 patients. *Surg Laparosc Endosc* 1996; **6**: 205–209.

Romagnuolo J, Currie G. Noninvasive *vs.* selective invasive biliary imaging for acute biliary pancreatitis: an economic evaluation by using decision tree analysis. *Gastrointest Endosc* 2005; **61**: 86–97.

Romero J, Sanabria A, Angarita M, Varón JC. Cost-effectiveness of computed tomography and ultrasound in the diagnosis of appendicitis. *Biomedica* 2008; **28**: 139–147.

Shaligram A, Pallati P, Simorov A, Meyer A, Oleynikov D. Do you need a computed tomographic scan to evaluate suspected appendicitis in young men: an administrative database review. *Am J Surg* 2012; **204**: 1025–1030.

Sheffield KM, Ramos KE, Djukom CD, Jimenez CJ, Mileski WJ, Kimbrough TD *et al.* Implementation of a critical pathway for complicated gallstone disease: translation of population-based data into clinical practice. *J Am Coll Surg* 2011; **212**: 835–843.

Simorov A, Ranade A, Parcells J, Shaligram A, Shostrom V, Boilesen E *et al.* Emergent cholecystostomy is superior to open cholecystectomy in extremely ill patients with acalculous cholecystitis: a large multicenter outcome study. *Am J Surg* 2013; **206**: 935–941.

Singh H, Latosinsky S, Spiegel BM, Targownik LE. The cost-effectiveness of colonic stenting as a bridge to curative surgery in patients with acute left-sided malignant colonic obstruction: a Canadian perspective. *Can J Gastroenterol* 2006; **20**: 779–785.

Sporn E, Petroski GF, Mancini GJ, Astudillo JA, Miedema BW, Thaler K. Laparoscopic appendectomy: is it worth the cost? Trend analysis in the US from 2000 to 2005. *J Am Coll Surg* 2009; **208**: 179–185.

Tabone LE, Conlon M, Fernando E, Yi S, Sarker S, Fisichella PM *et al.* A practical cost-effective management strategy for gallstone pancreatitis. *Am J Surg* 2013; **206**: 472–477.

Targownik LE, Spiegel BM, Sack J, Hines OJ, Dulai GS, Gralnek IM *et al.* Colonic stent *vs.* emergency surgery for management of acute left-sided malignant colonic obstruction: a decision analysis. *Gastrointest Endosc* 2004; **60**: 865–874.

Tiwari MM, Reynoso JF, Tsang AW, Oleynikov D. Comparison of outcomes of laparoscopic and open appendectomy in management of uncomplicated and complicated appendicitis. *Ann Surg* 2011; **254**: 927–932.

Tsoi KK, Lau JY, Sung JJ. Cost-effectiveness analysis of high-dose omeprazole infusion before endoscopy for patients with upper-GI bleeding. *Gastrointest Endosc* 2008; **67**: 1056–1063.

Tsugawa K, Koyanagi N, Hashizume M, Tomikawa M, Ayukawa K, Akahoshi K *et al.* A comparison of an open and laparoscopic appendectomy for patients with liver cirrhosis. *Surg Laparosc Endosc Percutan Tech* 2001; **11**: 189–194.

Turhan AN, Kapan S, Kütükçü E, Yiǧitbaş H, Hatipoǧlu S, Aygün E. Comparison of operative and non operative management of acute appendicitis. *Turk J Trauma Emerg Surg* 2009; **15**: 459–462.

Varadarajulu S, Roy A, Lopes T, Drelichman ER, Kim M. Endoscopic stenting *versus* surgical colostomy for the management of malignant colonic obstruction: comparison of hospital costs and clinical outcomes. *Surg Endosc Other Intervent Tech* 2011; **25**: 2203–2209.

Varela JE, Hinojosa MW, Nguyen NT. Laparoscopy should be the approach of choice for acute appendicitis in the morbidly obese. *Am J Surg* 2008; **196**: 218–222.

Wilson E, Gurusamy K, Gluud C, Davidson BR. Cost–utility and value-of-information analysis of early *versus* delayed laparoscopic cholecystectomy for acute cholecystitis. *Br J Surg* 2010; **97**: 210–219.

Wu HS, Lai HW, Kuo SJ, Lee YT, Chen DR, Chi CW *et al.* Competitive edge of laparoscopic appendectomy *versus* open appendectomy: a subgroup comparison analysis. *J Laparoendosc Adv Surg Tech A* 2011; **21**: 197–202.

Xinopoulos D, Dimitroulopoulos D, Theodosopoulos T, Tsamakidis K, Bitsakou G, Plataniotis G *et al.* Stenting or stoma creation for patients with inoperable malignant colonic obstructions: results of a study and cost-effectiveness analysis. *Surg Endosc* 2004; **18**: 421–426.

Xinopoulos D, Dimitroulopoulos D, Theodosopoulos T, Tsamakidis K, Paraskevas I, Vassilopoulos P *et al.* Palliation of inoperable malignant colonic obstruction: comparison and cost effectiveness analysis between stent placement and stoma creation. *Ann Gastroenterol* 2004; **17**: 294–299.

Yang P, Feng KX, Luo H, Wang D, Hu ZH. Acute biliary pancreatitis treated by early endoscopic intervention. *Panminerva Med* 2012; **54**: 65–69.

Yeh CC, Wu SC, Liao CC, Su LT, Hsieh CH, Li TC. Laparoscopic appendectomy for acute appendicitis is more favorable for patients with comorbidities, the elderly, and those with complicated appendicitis: a nationwide population-based study. *Surg Endosc* 2011; **25**: 2932–2942.

Zaninotto G, Rossi M, Anselmino M, Costantini M, Pianalto S, Baldan N *et al.* Laparoscopic *versus* conventional surgery for suspected appendicitis in women. *Surg Endosc* 1995; **9**: 337–340.

Zhao X, Chen DZ, Lang R, Jin ZK, Fan H, Wu TM *et al.* Enhanced recovery in the management of mild gallstone pancreatitis: a prospective cohort study. *Surg Today* 2013; **43**: 643–647.

Zhu B, Zhang Z, Wang Y, Gong K, Lu Y, Zhang N. Comparison of laparoscopic cholecystectomy for acute cholecystitis within and beyond 72 h of symptom onset during emergency admissions. *World J Surg* 2012; **36**: 2654–2658.

**Not a systematic review**

Bansal VK, Misra MC, Kumar S, Rao YK, Singhal P, Goswami A *et al.* A prospective randomized study comparing suture mesh fixation *versus* tacker mesh fixation for laparoscopic repair of incisional and ventral hernias. *Surg Endosc* 2011; **25**: 1431–1438.

Burch JM, Franciose RJ, Moore EE, Biffl WL, Offner PJ. Single-layer continuous *versus* two-layer interrupted intestinal anastomosis: a prospective randomized trial. *Ann Surg* 2000; **231**: 832–837.

Canto MI, Chak A, Stellato T, Sivak MV Jr. Endoscopic ultrasonography *versus* cholangiography for the diagnosis of choledocholithiasis. *Gastrointest Endosc* 1998; **47**: 439–448.

Chang L, Lo S, Stabile BE, Lewis RJ, Toosie K, de Virgilio C. Preoperative *versus* postoperative endoscopic retrograde cholangiopancreatography in mild to moderate gallstone pancreatitis: a prospective randomized trial. *Ann Surg* 2000; **231**: 82–87.

Cheng Y, Zhou R, Wu S, Lu J, Xiong X, Lin Y *et al.* Abdominal drainage after appendectomy for complicated appendicitis. (Protocol) *Cochrane Database Syst Rev* 2012; (10)CD010168.

Cirocchi R, Farinella E, Trastulli S, Boselli C, Montedori A, Gullà N *et al.* Laparoscopic *versus* open surgery for colonic diverticulitis. (Protocol) *Cochrane Database Syst Rev* 2011; (8)CD009277.

Csikesz NG, Tseng JF, Shah SA. Trends in surgical management for acute cholecystitis. *Surgery* 2008; **144**: 283–289.

Dickinson K, McCormack K, Scott N, Fawole A, White C, Grant AM. Mesh fixation techniques for laparoscopic inguinal hernia repair in adults. (Protocol) *Cochrane Database Syst Rev* 2011; (1)CD008954.

Dutta AK, Goel A, Kirubakaran R, Chacko A. Nasogastric *versus* nasojejunal tube feeding for severe acute pancreatitis. (Protocol) *Cochrane Database Syst Rev* 2013; (6)CD010582.

ECRI Institute. *Magnetic Resonance Cholangiopancreatography for Diagnosis of Bile Duct Stones*. ECRI Institute: Plymouth Meeting, 2003: 36.

Englert ZP, White MA, Fitzgerald TL, Vadlamudi A, Zervoudakis G, Zervos EE. Surgical management of malignant bowel obstruction: at what price palliation? *Am Surg* 2012; **78**: 647–652.

Friedrich M, Mueller-Riemenschneider FM, Roll S, Kulp W, Vauth C, Greiner W *et al.* *Vergleich der laparoskopischen narbenhernioplastik und der konventionellen operation mit und ohne netzeinlage – effektivitaet und kostennutzenrelation.* [*Health Technology Assessment of Laparoscopic Compared to Conventional Surgery With and Without Mesh for Incisional Hernia Repair Regarding Safety, Efficacy and Cost-Effectiveness*]. HTA-Bericht 67German Agency for Health Technology Assessment at the German Institute for Medical Documentation and Information: Cologne,2008. <https://portal.dimdi.de/de/hta/hta_berichte/hta143_bericht_de.pdf> [accessed 27 January 2017]

Gholipour C, Shalchi RA, Abassi M. Efficacy and safety of early laparoscopic common bile duct exploration as primary procedure in acute cholangitis caused by common bile duct stones. *J Laparoendosc Adv Surg Tech* *A* 2007; **17**: 634–638.

Glavic Z, Begic L, Simlesa D, Rukavina A. Treatment of acute cholecystitis: a comparison of open *vs* laparoscopic cholecystectomy. *Surg Endosc* 2001; **15**: 398–401.

Gurusamy K, Wilson E, Burroughs AK, Davidson BR. Intra-operative *vs* pre-operative endoscopic sphincterotomy in patients with gallbladder and common bile duct stones: cost-utility and value-of-information analysis. *Appl Health Econ Health Policy* 2012; **10**: 15–29.

Heikkinen T, Haukipuro K, Leppälä J, Hulkko A. Total costs of laparoscopic and Lichtenstein inguinal hernia repairs. *Surg Laparosc Endosc* 1997; **7**: 1–5.

Heili MJ, Wintz NK, Fowler DL. Choledocholithiasis: endoscopic *versus* laparoscopic management. *Am Surg* 1999; **65**: 135–138.

Johnson AB, Peetz ME. Laparoscopic appendectomy is an acceptable alternative for the treatment of perforated appendicitis. *Surg Endosc* 1998; **12**: 940–943.

Kuwabara K, Matsuda S, Fushimi K, Ishikawa KB, Horiguchi H, Fujimori K. Relationships of age, cholecystectomy approach and timing with the surgical and functional outcomes of elderly patients with cholecystitis. *Int J Surg* 2011; **9**: 392–399.

Kuwabara K, Matsuda S, Fushimi K, Ishikawa KB, Horiguchi H, Fujimori K *et al.* Impact of timing of cholecystectomy and bile duct interventions on quality of cholecystitis care. *Int J Surg* 2009; **7**: 243–249.

Langeveld HR, van’t Riet M, Weidema WF, Stassen LP, Steyerberg EW, Lange J *et al.* Total extraperitoneal inguinal hernia repair compared with Lichtenstein (the LEVEL-Trial): a randomized controlled trial. *Ann Surg* 2010; **251**: 819–824.

Leida Z, Ping B, Shuguang W, Yu H. A randomized comparison of primary closure and T-tube drainage of the common bile duct after laparoscopic choledochotomy. *Surg Endosc* 2008; **22**: 1595–1600.

Ma L, Wang T, Ma B, Liu Y. Octreotide for inoperable malignant bowel obstruction. (Protocol) *Cochrane Database Syst Rev* 2010; (3)CD008396.

Mauro DD, Faraci R, Mariani L, Cudazzo E, Costi R. Rendezvous technique for cholecystocholedochal lithiasis in octogenarians: is it as effective as in younger patients, or should endoscopic sphincterotomy followed by laparoscopic cholecystectomy be preferred? *J Laparoendosc Adv Surg Tech A* 2014; **24**: 13–21.

Molina Linde JM, Villegas PR, Lacalle R Jr, Parra MP, Diaz GD, Gomez BL *et al.* Estandares de uso adecuado de tecnologias sanitarias (Metodo RAND) 2. Laparoscopia en colecistitis aguda, coledocolitiasis y colelitiasis. [*Standards for Health Technologies Appropriateness: Laparoscopy in Acute Cholecystitis, Choledocholithiasis and Cholelithiasis. Development of Criteria for Appropriateness*]. Andalusian Agency for Health Technology Assessment (AETSA): Seville, 2008.

Morino M, Baracchi F, Miglietta C, Furlan N, Ragona R, Garbarini A. Preoperative endoscopic sphincterotomy *versus* laparoendoscopic rendezvous in patients with gallbladder and bile duct stones. *Ann Surg* 2006; **244**: 889–893.

Moya P, Arroyo A, Pérez-Legaz J, Serrano P, Candela F, Soriano-Irigaray L *et al.* Applicability, safety and efficiency of outpatient treatment in uncomplicated diverticulitis. *Tech Coloproctol* 2012; **16**: 301–307.

National Institute for Clinical Excellence (NICE). *Guidance on the Use of Laparoscopic Surgery for Inguinal Hernia*. NICE: London, 2001.

National Institute for Clinical Excellence (NICE). *Percutaneous Pancreatic Necrosectomy.* NICE: London, 2003.

National Institute for Clinical Excellence (NICE). *Laparoscopic Surgery for Inguinal Hernia Repair.* NICE: London, 2004.

National Institute for Health and Clinical Excellence (NICE*). Infliximab for the Treatment of Acute Exacerbations of Ulcerative Colitis.* NICE: London, 2008.

National Institute for Health and Clinical Excellence (NICE). *Single-Incision Laparoscopic Cholecystectomy*. NICE: London, 2010.

Nguyen NT, Zainabadi K, Mavandadi S, Paya M, Stevens CM, Root J *et al.* Trends in utilization and outcomes of laparoscopic *versus* open appendectomy. *Am J Surg* 2004; **188**: 813–820.

Ogata M, Mateer JR, Condon RE. Prospective evaluation of abdominal sonography for the diagnosis of bowel obstruction. *Ann Surg* 1996; **223**: 237–241.

Park SH, Kang CM, Chae YS, Kim KS, Choi JS, Lee WJ *et al.* Percutaneous cholecystostomy using a central venous catheter is effective for treating high-risk patients with acute cholecystitis. *Surg Laparosc Endosc Percutan Tech* 2005; **15**: 202–208.

Peterson WL, Cook DJ. Antisecretory therapy for bleeding peptic ulcer. *JAMA* 1998; **280**: 877–878.

Pokala N, Delaney CP, Senagore AJ, Brady KM, Fazio VW. Laparoscopic *vs* open total colectomy: a case-matched comparative study. *Surg Endosc* 2005; **19**: 531–535.

Prieto-Díaz-Chávez E, Medina-Chávez JL, Anaya-Prado R. A cost-effectiveness analysis of tension-free *versus* shouldice inguinal hernia repair: a randomized double-blind clinical trial. *Hernia* 2009; **13**: 233–238.

Purins A, Merlin T, Mundy L, Hiller JE. *Infrared Spectroscopy for the Diagnosis of Acute Pancreatitis*. Horizon Scanning Prioritising Summary Volume 19. Adelaide Health Technology Assessment (AHTA): Adelaide, 2008. <http://www.horizonscanning.gov.au/internet/horizon/publishing.nsf/Content/BB580B674729F620CA2575AD0080F351/$File/Volume_19_Feb_2008_IRS.pdf> [accessed 27 January 2017].

Rea JD, Herzig DO, Diggs BS, Cone MM, Lu KC. Use and outcomes of emergent laparoscopic resection for acute diverticulitis. *Am J Surg* 2012; **203**: 639–643.

Rogers SJ, Cello JP, Horn JK, Siperstein AE, Schecter WP, Campbell AR *et al.* Prospective randomized trial of LC+LCBDE vs ERCP/S+LC for common bile duct stone disease. *Arch Surg* 2010; **145**: 28–33.

Saba L, Mallarini G. Spiral computed tomography imaging of bowel ischemia: a literature review. *Panminerva Med* 2007; **49**: 35–41.

Sajid M, Leaver C, Sains P, Baig MK. Lightweight versus Heavyweight mesh for laparoscopic repair of inguinal hernia. (Protocol) *Cochrane Database Syst Rev* 2011; (12)CD009475.

Shekherdimian S, DeUgarte D. Transumbilical laparoscopic-assisted appendectomy: an extracorporeal single-incision alternative to conventional laparoscopic techniques. *Am Surg* 2011; **77**: 557–560.

Shinohara T, Yamashita Y, Naito M, Maki K, Hashimoto T, Matsuo K *et al.* Prospective randomized trial of a closed-suction drain *versus* a Penrose drain after a colectomy. *Hepatogastroenterology* 2010; **57**: 1119–1122.

St Peter SD, Tsao K, Spilde TL, Holcomb GW III, Sharp SW, Murphy JP *et al.* Single daily dosing ceftriaxone and metronidazole *vs* standard triple antibiotic regimen for perforated appendicitis in children: a prospective randomized trial. *J Pediatr Surg* 2008; **43**: 981–985.

Takegami K, Kawaguchi Y, Nakayama H, Kubota Y, Nagawa H. Impact of a clinical pathway and standardization of treatment for acute appendicitis. *Surg Today* 2003; **33**: 336–341.

van der Zwaal P, van den Berg IR, Plaisier PW, Tutein Nolthenius RP. Mesh fixation using staples in Lichtenstein's inguinal hernioplasty: fewer complications and fewer recurrences. *Hernia* 2008; **12**: 391–394.

Veedfald S, Penninga L, Wettergren A, Gluud C. Bile acids for biliary colic. (Protocol) *Cochrane Database Syst Rev* 2011; (8)CD009253.

Wei Q, Wang JG, Li LB, Li JD. Management of choledocholithiasis: comparison between laparoscopic common bile duct exploration and intraoperative endoscopic sphincterotomy. *World J Gastroenterol* 2003; **9**: 2856–2858.

Wellwood J, Sculpher MJ, Stoker D, Nicholls GJ, Geddes C, Whitehead A *et al.* Randomised controlled trial of laparoscopic *versus* open mesh repair for inguinal hernia: outcome and cost. *BMJ* 1998; **317**: 103–110.

Wills VL, Jorgensen JO, Hunt DR. A randomized controlled trial comparing cholecystocholangiography with cystic duct cholangiography during laparoscopic cholecystectomy. *ANZ J Surg* 2000; **70**: 573–577.

**Elective/non-emergency care**

Ahmad NZ, Byrnes G, Naqvi SA. A meta-analysis of ambulatory *versus* inpatient laparoscopic cholecystectomy. *Surg Endosc* 2008; **22**: 1928–1934.

Akshintala VS, Hutfless SM, Colantuoni E, Kim KJ, Khashab MA, Li T *et al.* Systematic review with network meta-analysis: pharmacological prophylaxis against post-ERCP pancreatitis. *Aliment Pharmacol Ther* 2013; **38**: 1325–1337.

Alexakis N, Connor S. Meta-analysis of one- *vs.* two-stage laparoscopic/endoscopic management of common bile duct stones. *HPB* 2012; **14**: 254–259.

Aly O, Green A, Joy M, Wong CH, Al-Kandari A, Cheng S *et al.* Is laparoscopic inguinal hernia repair more effective than open repair? *J Coll Physicians Surg Pak* 2011; **21**: 291–296.

Amato B, Moja L, Panico S, Persico G, Rispoli C, Rocco N *et al.* Shouldice technique *versus* other open techniques for inguinal hernia repair. *Cochrane Database Syst Rev* 2012; (4)CD001543.

Ammori BJ, Birbas K, Davides D, Vezakis A, Larvin M, McMahon MJ. Routine *vs* 'on demand' postoperative ERCP for small bile duct calculi detected at intraoperative cholangiography: clinical evaluation and cost analysis. *Surg Endosc* 2000; **14**: 1123–1126.

Anderson G, Boldiston C, Woods S, O’Brien P. A cost-effectiveness evaluation of 3 antimicrobial regimens for the prevention of infective complications after abdominal surgery. *Arch Surg* 1996; **131**: 744–748.

Antoniou SA, Pointner R, Granderath FA. Single-incision laparoscopic cholecystectomy: a systematic review. *Surg Endosc* 2011; **25**: 367–377.

Aronson N, Flamm CR, Mark D, Lefevre F, Bohn RL, Finkelstein B. *Endoscopic Retrograde Cholangiopancreatography*. Evidence Report/Technology Assessment No. 50. Agency for Healthcare Research and Quality (AHRQ): Rockville, 2001.

Ayub K, Slavin J, Imada R. Endoscopic retrograde cholangiopancreatography in gallstone-associated acute pancreatitis. *Cochrane Database Syst Rev* 2010; (1)CD003630.

Bachmann K, Krause G, Rawnaq T, Tomkotter L, Vashist Y, Shahmiri S *et al.* Impact of early or delayed elective resection in complicated diverticulitis. *World J Gastroenterol* 2011; **17**: 5274–5279.

Bakota B, Kopljar M, Patrlj L, Franic M. Anaesthetic techniques for open inguinal and femoral hernia repair in adults. *Cochrane Database Syst Rev* 2012; (2)CD006684.

Beets GL, Dirksen CD, Go PM, Geisler FE, Baeten CG, Kootstra G. Open or laparoscopic preperitoneal mesh repair for recurrent inguinal hernia? a randomised controlled trial. *Surg Endosc* 1999; **13**: 323–327.

Biondo S, Golda T, Kreisler E, Espin E, Vallribera F, Oteiza F *et al.* Outpatient *versus* hospitalization management for uncomplicated diverticulitis: a prospective, multicenter randomized clinical trial (DIVER trial). *Ann Surg* 2014; **259**: 38–44.

Bucher P, Pugin F, Buchs NC, Ostermann S, Morel P. Randomized clinical trial of laparoendoscopic single-site *versus* conventional laparoscopic cholecystectomy. *Br J Surg* 2011; **98**: 1695–1702.

Carter F, Thabane M, Alsayb M, Marshall JK. Mesalamine (5-ASA) for the management of diverticulitis. *Cochrane Database Syst Rev* 2012; (5)CD009839.

Chung RS, Rowland DY. Meta-analyses of randomized controlled trials of laparoscopic *vs* conventional inguinal hernia repairs. *Surg Endosc* 1999; **13**: 689–694.

Clayton ES, Connor S, Alexakis N, Leandros E. Meta-analysis of endoscopy and surgery *versus* surgery alone for common bile duct stones with the gallbladder in situ. *Br J Surg* 2006; **93**: 1185–1191.

Colli A, Conte D, Della VS, Sciola V, Fraquelli M. Meta-analysis: nonsteroidal anti-inflammatory drugs in biliary colic. *Aliment Pharmacol Ther* 2012; **35**: 1370–1378.

Dasari BVM, Tan CJ, Gurusamy KS, Martin DJ, Kirk G, McKie L *et al.* Surgical *versus* endoscopic treatment of bile duct stones. *Cochrane Database Syst Rev* 2013; (12)CD003327.

Ibañez Rde M, Nahban Al Saied SA, Alonso VJ, Rodriguez Canales JM, Blanco PC, Escribano SF. Cost-effectiveness of primary abdominal wall hernia repair in a 364-bed provincial hospital of Spain. *Hernia* 2011; **15**: 377–385.

Dirksen CD, Ament AJ, Adang EM, Beets GL, Go PM, Baeten CG *et al.* Cost-effectiveness of open *versus* laparoscopic repair for primary inguinal hernia. *Int J Tech Assess Health Care* 1998; **14**: 472–483.

Dobson MW, Geisler D, Fazio V, Remzi F, Hull T, Vogel J. Minimally invasive surgical wound infections: laparoscopic surgery decreases morbidity of surgical site infections and decreases the cost of wound care. *Colorectal Dis* 2011; **13**: 811–815.

Downs SH, Black NA, Devlin HB, Royston CM, Russell RC. Systematic review of the effectiveness and safety of laparoscopic cholecystectomy. *Ann R Coll Surg Engl* 1996; **78**: 241–323.

Duepree HJ, Senagore AJ, Delaney CP, Brady KM, Fazio VW. Advantages of laparoscopic resection for ileocecal Crohn,'s disease. *Dis Colon Rectum* 2002; **45**: 605–610.

Eisenberg DP, Wey J, Bao PQ, Saul M, Watson AR, Schraut WH *et al.* Short- and long-term costs of laparoscopic colectomy are significantly less than open colectomy. *Surg Endosc* 2010; **24**: 2128–2134.

EU Hernia Trialists Collaboration. Laparoscopic compared with open methods of groin hernia repair: systematic review of randomized controlled trials. *Br J Surg* 2000; **87**: 860–867.

EUHernia Trialists Collaboration. Mesh compared with non-mesh methods of open groin hernia repair: systematic review of randomized controlled trials. *Br J Surg* 2000; **87**: 854–859.

Feuer DJ, Broadley KE. Corticosteroids for the resolution of malignant bowel obstruction in advanced gynaecological and gastrointestinal cancer. *Cochrane Database Syst Rev* 1999: (3)CD001219.

Ford JA, Soop M, Du J, Loveday BP, Rodgers M. Systematic review of intraoperative cholangiography in cholecystectomy. *Br J Surg* 2012; **99**: 160–167.

Fullum TM, Ladapo JA, Borah BJ, Gunnarsson CL. Comparison of the clinical and economic outcomes between open and minimally invasive appendectomy and colectomy: evidence from a large commercial payer database. *Surg Endosc Other Intervent Tech* 2010; **24**: 845–853.

Güenaga KF, Lustosa SAS, Saad SS, Saconato H, Matos D. Ileostomy or colostomy for temporary decompression of colorectal anastomosis. *Cochrane Database Syst Rev* 2007; (1)CD004647.

Gaertner WB, Kwaan MR, Madoff RD, Willis D, Belzer GE, Rothenberger DA *et al.* The evolving role of laparoscopy in colonic diverticular disease: a systematic review. *World J Surg* 2013; **37**: 629–638.

Gao M, Han J, Tian J, Yang K. Vypro II mesh for inguinal hernia repair: a meta analysis of randomized controlled trials. *Ann Surg* 2010; **251**: 838–842.

Gervaz P, Mugnier-Konrad B, Morel P, Huber O, Inan I. Laparoscopic *versus* open sigmoid resection for diverticulitis: long-term results of a prospective, randomized trial. *Surg Endosc* 2011; **25**: 3373–3378.

Gholghesaei M, Langeveld HR, Veldkamp R, Bonjer HJ. Costs and quality of life after endoscopic repair of inguinal hernia vs open tension-free repair: a review. *Surg Endosc* 2005; **19**: 816–821.

Gong K, Zhang N, Lu Y, Zhu B, Zhang Z, Du D *et al.* Comparison of the open tension-free mesh-plug, transabdominal preperitoneal (TAPP), and totally extraperitoneal (TEP) laparoscopic techniques for primary unilateral inguinal hernia repair: a prospective randomized controlled trial. *Surg Endosc* 2011; **25**: 234–239.

Grant AM; Eu Hernia Trialists Collaboration. Laparoscopic *versus* open groin hernia repair: meta-analysis of randomised trials based on individual patient data. *Hernia* 2002; **6**: 2–10.

Grant AM, Go P, Fingerhut A, Kingsnorth A, Merello J, O’Dwyer P *et al.* Repair of groin hernia with synthetic mesh: meta-analysis of randomized controlled trials. *Ann Surg* 2002; **235**: 322–332.

Gurusamy KS, Bong JJ, Fusai G, Davidson BR. Methods of cystic duct occlusion during laparoscopic cholecystectomy. *Cochrane Database Syst Rev* 2010; (10)CD006807.

Gurusamy KS, Samraj K. Primary closure *versus* T-tube drainage after open common bile duct exploration. *Cochrane Database Syst Rev* 2007: (1)CD005640.

Gurusamy KS, Samraj K, Davidson BR. Low pressure *versus* standard pressure pneumoperitoneum in laparoscopic cholecystectomy. *Cochrane Database Syst Rev* 2009; (2)CD006930.

Gurusamy KS, Tapuria N, Davidson BR. Methods of gallbladder dissection for laparoscopic cholecystectomy. *Cochrane Database Syst Rev* 2008; (2)CD007054.

Hodgson NC, Malthaner RA, Ostbye T. The search for an ideal method of abdominal fascial closure: a meta-analysis. *Ann Surg* 2000; **231**: 436–442.

Ishikawa M, Nakagawa T, Nishioka M, Ogata S, Miyauchi T, Kashiwagi Y *et al.* Costs and benefits of laparoscopic cholecystectomy: abdominal wall lifting *vs* pneumoperitoneum procedure. *Hepatogastroenterology* 2006; **53**: 497–500.

Joseph S, Moore BT, Sorensen GB, Earley JW, Tang F, Jones P *et al.* Single-incision laparoscopic cholecystectomy: a comparison with the gold standard. *Surg Endosc* 2011; **25**: 3008–3015.

Kald A, Anderberg B, Carlsson P, Park PO, Smedh K. Surgical outcome and cost-minimisation-analyses of laparoscopic and open hernia repair: a randomised prospective trial with one year follow up. *Eur J Surg* 1997; **163**: 505–510.

Kaltenthaler E, Vergel YB, Chilcott J, Thomas S, Blakeborough T, Walters SJ *et al.* A systematic review and economic evaluation of magnetic resonance cholangiopancreatography compared with diagnostic endoscopic retrograde cholangiopancreatography. *Health Technol Assess* 2004; **8**: No. 10: 1–102.

Karthikesalingam A, Markar SR, Holt PJ, Praseedom RK. Meta-analysis of randomized controlled trials comparing laparoscopic with open mesh repair of recurrent inguinal hernia. *Br J Surg* 2010; **97**: 4–11.

Kaul A, Hutfless S, Le H, Hamed SA, Tymitz K, Nguyen H *et al.* Staple *versus* fibrin glue fixation in laparoscopic total extraperitoneal repair of inguinal hernia: a systematic review and meta-analysis. *Surg Endosc* 2012; **26**: 1269–1278.

Lai HW, Loong CC, Wu CW, Lui WY. Watchful waiting *versus* interval appendectomy for patients who recovered from acute appendicitis with tumor formation: a cost-effectiveness analysis. *J Chin Med Assoc* 2005; **68**: 431–434.

Leontiadis GI, Sreedharan A, Dorward S, Barton P, Delaney B, Howden CW *et al.* Systematic reviews of the clinical effectiveness and cost-effectiveness of proton pump inhibitors in acute upper gastrointestinal bleeding: *H. pylori* eradication therapy. *Health Technol Assess* 2007; **11**: 67–74. [Other eligible chapters from the same report have been included]

Leontiadis GI, Sreedharan A, Dorward S, Barton P, Delaney B, Howden CW *et al.* Systematic reviews of the clinical effectiveness and cost-effectiveness of proton pump inhibitors in acute upper gastrointestinal bleeding: prophylactic PPI therapy. *Health Technol Assess* 2007; **11**: 75–97. [Other eligible chapters from the same report have been included]

Leontiadis GI, Sreedharan A, Dorward S, Barton P, Delaney B, Howden CW *et al.* Systematic reviews of the clinical effectiveness and cost-effectiveness of proton pump inhibitors in acute upper gastrointestinal bleeding: *H. pylori* eradication *versus* proton pump inhibitors. *Health Technol Assess* 2007; **11**: 139–144. [Other eligible chapters from the same report have been included.]

Liao WC, Tu YK, Wu MS, Wang HP, Lin JT, Leung JW *et al.* Balloon dilation with adequate duration is safer than sphincterotomy for extracting bile duct stones: a systematic review and meta-analyses. *Clin Gastroenterol Hepatol* 2012; **10**: 1101–1109.

Liberman MA, Phillips EH, Carroll BJ, Fallas M, Rosenthal R. Laparoscopic colectomy vs traditional colectomy for diverticulitis: outcome and costs. *Surg Endosc* 1996; **10**: 15–18.

Liberman MA, Phillips EH, Carroll BJ, Fallas MJ, Rosenthal R, Hiatt J. Cost-effective management of complicated choledocholithiasis: laparoscopic transcystic duct exploration or endoscopic sphincterotomy. *J Am Coll Surg* 1996; **182**: 488–494.

Liu WH, Yang B, Xu J. Stapled *versus* unstapled mesh in laparoscopic inguinal hernia repair: a systematic review. *Acad J Second Mil Med Univ* 2010; **31**: 1337–1340.

Liu Y, Su P, Lin S, Xiao K, Chen P, An S *et al.* Endoscopic papillary balloon dilatation *versus* endoscopic sphincterotomy in the treatment for choledocholithiasis: a meta-analysis. *J Gastroenterol Hepatol* 2012; **27**: 464–471.

Liu Y, Su P, Lin Y, Lin S, Xiao K, Chen P *et al.* Endoscopic sphincterotomy plus balloon dilation *versus* endoscopic sphincterotomy for choledocholithiasis: a meta-analysis. *J Gastroenterol Hepatol* 2013; **28**: 937–945.

Lustosa SA, Matos D, Atallah AN, Castro AA. Stapled *versus* handsewn methods for colorectal anastomosis surgery: a systematic review of randomized controlled trials. *Rev Paul Med* 2002; **120**: 132–136.

Maconi G, Barbara G, Bosetti C, Cuomo R, Annibale B. Treatment of diverticular disease of the colon and prevention of acute diverticulitis: a systematic review. *Dis Colon Rectum* 2011; **54**: 1326–1338.

Mancini GJ, Petroski GF, Lin WC, Sporn E, Miedema BW, Thaler K. Nationwide impact of laparoscopic lysis of adhesions in the management of intestinal obstruction in the US. *J Am Coll Surg* 2008; **207**: 520–526.

Markar SR, Karthikesalingam A, Thrumurthy S, Muirhead L, Kinross J, Paraskeva P. Single-incision laparoscopic surgery (SILS) *vs.* conventional multiport cholecystectomy: systematic review and meta-analysis. *Surg Endosc* 2012; **26**: 1205–1213.

Mavros MN, Mitsikostas PK, Alexiou VG, Peppas G, Falagas ME. Antimicrobials as an adjunct to pilonidal disease surgery: a systematic review of the literature. *Eur J Clin Microbiol Infect Dis* 2013; **32**: 851–858.

McCloy R, Randall D, Schug SA, Kehlet H, Simanski C, Bonnet F *et al.* Is smaller necessarily better? A systematic review comparing the effects of minilaparoscopic and conventional laparoscopic cholecystectomy on patient outcomes. *Surg Endosc* 2008; **22**: 2541–2553.

McCormack K, Scott N, Go PMNYH, Ross SJ, Grant A; EU Trialists Collaboration. Laparoscopic techniques *versus* open techniques for inguinal hernia repair. *Cochrane Database Syst Rev* 2003; (1)CD001785.

McCormack K, Wake B, Perez J, Fraser C, Cook J, McIntosh E *et al.* Laparoscopic surgery for inguinal hernia repair: systematic review of effectiveness and economic evaluation. *Health Technol Assess* 2005; **9**: 1–203.

McCormack K, Wake BL, Fraser C, Vale L, Perez J, Grant A. Transabdominal pre-peritoneal (TAPP) *versus* totally extraperitoneal (TEP) laparoscopic techniques for inguinal hernia repair: a systematic review. *Hernia* 2005; **9**: 109–114.

McKellar DP, Johnson RM, Dutro JA, Mellinger J, Bernie WA, Peoples JB. Cost-effectiveness of laparoscopic cholecystectomy. *Surg Endosc* 1995; **9**: 158–163.

Nilsson E, Ros A, Rahmqvist M, Backman K, Carlsson P. Cholecystectomy: costs and health-related quality of life – a comparison of two techniques. *Int J Qual Health Care* 2004; **16**: 473–482.

Panés J, Bouzas R, Chaparro M, García-Sánchez V, Gisbert JP, Martínez de Guereñu B *et al.* Systematic review: the use of ultrasonography, computed tomography and magnetic resonance imaging for the diagnosis, assessment of activity and abdominal complications of Crohn’'s disease. *Aliment Pharmacol Ther* 2011; **34**: 125–145.

Petrov MS, Uchugina AF, Kukosh MV. Does endoscopic retrograde cholangiopancreatography reduce the risk of local pancreatic complications in acute pancreatitis? A systematic review and metaanalysis. *Surg Endosc* 2008; **22**: 2338–2343.

Poulose BK, Speroff T, Holzman MD. Optimizing choledocholithiasis management: a cost-effectiveness analysis. *Arch Surg* 2007; **142**: 43–48.

Pu YW, Xing CG, Khan I, Zhao K, Zhu BS, Wu Y. Fistula plug *versus* conventional surgical treatment for anal fistulas: a systematic review and meta-analysis. *Saudi Med J* 2012; **33**: 962–966.

Purkayastha S, Tilney HS, Darzi AW, Tekkis PP. Meta-analysis of randomized studies evaluating chewing gum to enhance postoperative recovery following colectomy. *Arch Surg* 2008; **143**: 788–793.

Rábago LR, Vicente C, Soler F, Delgado M, Moral I, Guerra I *et al.* Two-stage treatment with preoperative endoscopic retrograde cholangiopancreatography (ERCP) compared with single-stage treatment with intraoperative ERCP for patients with symptomatic cholelithiasis with possible choledocholithiasis. *Endoscopy* 2006; **38**: 779–786.

Regge D, Hassan C, Pickhardt PJ, Laghi A, Zullo A, Kim DH *et al.* Impact of computer-aided detection on the cost-effectiveness of CT colongraphy. *Radiology* 2009; **250**: 488–497.

Rodríguez-Cerrillo M, Poza-Montoro A, Fernandez-Diaz E, Matesanz-David M, Iñurrieta Romero A. Treatment of elderly patients with uncomplicated diverticulitis, even with comorbidity, at home. *Eur J Intern Med* 2013; **24**: 430–432.

Rondelli F, Trastulli S, Avenia N, Schillaci G, Cirocchi R, Gulla N *et al.* Is laparoscopic right colectomy more effective than open resection? A meta-analysis of randomized and nonrandomized studies. *Colorectal Dis* 2012; **14**: e447–e469.

Sahai AV, Mauldin PD, Marsi V, Hawes RH, Hoffman BJ. Bile duct stones and laparoscopic cholecystectomy: a decision analysis to assess the roles of intraoperative cholangiography, EUS, and ERCP. *Gastrointest Endosc* 1999; **49**: 334–343.

Sajid MS, Khan MA, Ray K, Cheek E, Baig MK. Needlescopic *versus* laparoscopic cholecystectomy: a meta-analysis. *ANZ J Surg* 2009; **79**: 437–442.

Sajid MS, Ladwa N, Kalra L, Hutson K, Sains P, Baig MK. A meta-analysis examining the use of tacker fixation *versus* no-fixation of mesh in laparoscopic inguinal hernia repair. *Int J Surg* 2012; **10**: 224–231.

Samaranayake CB, Luo C, Plank AW, Merrie AE, Plank LD, Bissett IP. Systematic review on ventral rectopexy for rectal prolapse and intussusception. *Colorectal Dis* 2010; **12**: 504–512.

Schmedt CG, Sauerland S, Bittner R. Comparison of endoscopic procedures *vs* Lichtenstein and other open mesh techniques for inguinal hernia repair: a meta-analysis of randomized controlled trials. *Surg Endosc* 2005; **19**: 188–199.

Schultz LS. Laparoscopic *vs* inguinal hernia repairs: outcomes and costs. *Surg Endosc*1995; **9**: 1307–1311.

Scott N, Go PMNYH, Graham P, McCormack K, Ross SJ, Grant AM. Open mesh *versus* non-mesh for groin hernia repair. *Cochrane Database Syst Rev* 2001; (3)CD002197.

Shea JA, Healey MJ, Berlin JA, Clarke JR, Malet PF, Staroscik RN *et al.* Mortality and complications associated with laparoscopic cholecystectomy: a meta-analysis. *Ann Surg* 1996; **224**: 609–620.

Shikata S, Noguchi Y, Fukui T. Early *versus* delayed cholecystectomy for acute cholecystitis: a meta-analysis of randomized controlled trials. *Surg Today* 2005; **35**: 553–560.

Siddiqui MR, Sajid MS, Nisar A, Ali H, Zaborszky A, Hasan F. A meta-analysis of outcomes after routine aspiration of the gallbladder during cholecystectomy. *Int Surg* 2011; **96**: 21–27.

Simillis C, Purkayastha S, Yamamoto T, Strong SA, Darzi AW, Tekkis PP. A meta-analysis comparing conventional end-to-end anastomosis *vs.* other anastomotic configurations after resection in Crohn’s disease. *Dis Colon Rectum* 2007; **50**: 1674–1687.

Simons MP, Kleijnen J, van Geldere D, Hoitsma HF, Obertop H. Role of the Shouldice technique in inguinal hernia repair: a systematic review of controlled trials and a meta-analysis. *Br J Surg* 1996; **83**: 734–738.

Srinivasa S, Kahokehr AA, Yu TC, Hill AG. Preoperative glucocorticoid use in major abdominal surgery: systematic review and meta-analysis of randomized trials. *Ann Surg* 2011; **254**: 183–191.

Srivastava A, Srinivas G, Misra MC, Pandav CS, Seenu V, Goyal A. Cost-effectiveness analysis of laparoscopic *versus* minilaparotomy cholecystectomy for gallstone disease: a randomized trial. *Int J Technol Assess Health Care* 2001; **17**: 497–502.

Stevens HP, van de Berg M, Ruseler CH, Wereldsma JC. Clinical and financial aspects of cholecystectomy: laparoscopic versus open technique. *World J Surg* 1997; **21**: 91–97.

Stylopoulos N, Gazelle GS, Rattner DW. A cost-utility analysis of treatment options for inguinal hernia in 1 513 008 adult patients. *Surg Endosc* 2003; **17**: 180–189.

Subramanian V, Pollok RC, Kang JY, Kumar D. Systematic review of postoperative complications in patients with inflammatory bowel disease treated with immunomodulators. *Br J Surg* 2006; **93**: 793–799.

Sun S, Yang K, Gao M, He X, Tian J, Ma B. Three-port *versus* four-port laparoscopic cholecystectomy: meta-analysis of randomized clinical trials. *World J Surg* 2009; **33**: 1904–1908.

Tam KW, Liang HH, Chai CY. Outcomes of staple fixation of mesh versus nonfixation in laparoscopic total extraperitoneal inguinal repair: a meta-analysis of randomized controlled trials. *World J Surg* 2010; **34**: 3065–3074.

Teoh AY, Cheung FK, Hu B, Pan YM, Lai LH, Chiu PW *et al.* Randomized trial of endoscopic sphincterotomy with balloon dilation *versus* endoscopic sphincterotomy alone for removal of bile duct stones. *Gastroenterology* 2013; **144**: 341–345.e341.

Tiwari MM, Reynoso JF, High R, Tsang AW, Oleynikov D. Safety, efficacy, and cost-effectiveness of common laparoscopic procedures. *Surg Endosc Other Intervent Tech* 2011; **25**: 1127–1135.

Tranchart H, Ketoff S, Lainas P, Pourcher G, Di Giuro G, Tzanis D *et al.* Single incision laparoscopic cholecystectomy: for what benefit? *HPB* 2013; **15**: 433–438.

Traverso LW, Roush TS, Koo K. CBD stones: outcomes and costs – laparoscopic transcystic techniques other than choledochoscopy. *Surg Endosc* 1995; **9**: 1242–1244.

Urbach DR, Khajanchee YS, Jobe BA, Standage BA, Hansen PD, Swanstrom LL. Cost-effective management of common bile duct stones: a decision analysis of the use of endoscopic retrograde cholangiopancreatography (ERCP), intraoperative cholangiography, and laparoscopic bile duct exploration. *Surg Endosc* 2001; **15**: 4–13.

Vale L, Grant A, McCormack K, Scott NW; EU Hernia Trialists Collaboration. Cost-effectiveness of alternative methods of surgical repair of inguinal hernia. *Int J Technol Assess Health Care* 2004; **20**: 192–200.

van der Voort M, Heijnsdijk EA, Gouma DJ. Bowel injury as a complication of laparoscopy. *Br J Surg* 2004; **91**: 1253–1258.

Varela JE, Asolati M, Huerta S, Anthony T. Outcomes of laparoscopic and open colectomy at academic centers. *Am J Surg* 2008; **196**: 403–406.

Villatoro E, Mulla M, Larvin M. Antibiotic therapy for prophylaxis against infection of pancreatic necrosis in acute pancreatitis. *Cochrane Database Syst Rev* 2010; (5)CD002941.

Wang B, Guo Z, Liu Z, Wang Y, Si Y, Zhu Y *et al.* Preoperative *versus* intraoperative endoscopic sphincterotomy in patients with gallbladder and suspected common bile duct stones: system review and meta-analysis. *Surg Endosc Other Intervent Tech* 2013; **27**: 2454–2465.

Weinberg B, Shindy W, Lo S. Endoscopic balloon sphincter dilation (sphincteroplasty) versus sphincterotomy for common bile duct stones. *Cochrane Database Syst Rev* 2006: (4)CD004890.

Wenner J, Graffner H, Lindell G. A financial analysis of laparoscopic and open cholecystectomy. *Surg Endosc* 1995; **9**: 702–705.

Yang XM, Hu B. Endoscopic sphincterotomy plus large-balloon dilation endoscopic sphincterotomy for choledocholithiasis: a meta-analysis. *World J Gastroenterol* 2013; **19**: 9453–9460.

Yassin NA, Hammond TM, Lunniss PJ, Phillips RK. Ligation of the intersphincteric fistula tract in the management of anal fistula: a systematic review. *Colorectal Dis* 2013; **15**: 527–535.

Yin Z, Xu K, Sun J, Zhang J, Xiao Z, Wang J *et al.* Is the end of the T-tube drainage era in laparoscopic choledochotomy for common bile duct stones is coming? A systematic review and meta-analysis. *Ann Surg* 2013; **257**: 54–66.

Zhao G, Gao P, Ma B, Tian J, Yang K. Open mesh techniques for inguinal hernia repair: a meta-analysis of randomized controlled trials. *Ann Surg* 2009; **250**: 35–42.

Zhao HC, He L, Zhou DC, Geng XP, Pan FM. Meta-analysis comparison of endoscopic papillary balloon dilatation and endoscopic sphincteropapillotomy. *World J Gastroenterol* 2013; **19**: 3883–3891.

Zingg U, Pasternak I, Guertler L, Dietrich M, Wohlwend KA, Metzger U. Early *vs.* delayed elective laparoscopic-assisted colectomy in sigmoid diverticulitis: timing of surgery in relation to the acute attack. *Dis Colon Rectum* 2007; **50**: 1911–1917.

Zwaal P, Berg IR, Plaisier PW, Tutein Nolthenius RP. Mesh fixation using staples in Lichtenstein’s inguinal hernioplasty: fewer complications and fewer recurrences. *Hernia* 2008; **12**: 391–394.

**Not an eligible condition**

Dutch Pancreatitis Study Group. A step-up approach or open necrosectomy for necrotizing pancreatitis. *N Engl J Med* 2010; **362**: 1491–1502.

Andriulli A, Leandro G, Clemente R, Festa V, Caruso N, Annese V *et al.* Meta-analysis of somatostatin, octreotide and gabexate mesilate in the therapy of acute pancreatitis. *Aliment Pharmacol Ther* 1998; **12**: 237–245.

Aoun E, Chen J, Reighard D, Gleeson FC, Whitcomb DC, Papachristou GI. Diagnostic accuracy of interleukin-6 and interleukin-8 in predicting severe acute pancreatitis: a meta-analysis. *Pancreatology* 2009; **9**: 777–785.

Bai M, Qi X, Yang M, Han G, Fan D. Combined therapies *versus* monotherapies for the first variceal bleeding in patients with high-risk varices: a meta-analysis of randomized controlled trials. *J Gastroenterol Hepatol* 2014; **29:** 442–452.

Bai Y, Gao J, Zou DW, Li ZS. Prophylactic antibiotics cannot reduce infected pancreatic necrosis and mortality in acute necrotizing pancreatitis: evidence from a meta-analysis of randomized controlled trials. *Am J Gastroenterol* 2008; **103**: 104–110.

Bañares R, Albillos A, Rincón D, Alonso S, González M, Ruiz-del-Arbol L *et al.* Endoscopic treatment *versus* endoscopic plus pharmacologic treatment for acute variceal bleeding: a meta-analysis. *Hepatology* 2002; **35**: 609–615.

Beenen E, Brown L, Connor S. A comparison of the hospital costs of open *vs.* minimally invasive surgical management of necrotizing pancreatitis. *HPB* 2011; **13**: 178–184.

Boele van Hensbroek P, Wind J, Dijkgraaf MG, Busch OR, Goslings JC. Temporary closure of the open abdomen: a systematic review on delayed primary fascial closure in patients with an open abdomen. *World J Surg* 2009; **33**: 199–207.

Abou-Assi S, Craig K, O’Keefe SJ. Hypocaloric jejunal feeding is better than total parenteral nutrition in acute pancreatitis: results of a randomized comparative study. *Am J Gastroenterol* 2002; **97**: 2255–2262.

Cao Y, Xu Y, Lu T, Gao F, Mo Z. Meta-analysis of enteral nutrition *versus* total parenteral nutrition in patients with severe acute pancreatitis. *Ann Nutr Metab* 2008; **53**: 268–275.

Castellanos Ríos E, Seron P, Gisbert JP, Bonfill Cosp X. Endoscopic injection of cyanoacrylate glue *versus* other endoscopic procedures for acute bleeding gastric varices in patients with portal hypertension. *Cochrane Database Syst Rev* 2012; (10)CD010180.

Chang DC, Wilson SE. Meta-analysis of the clinical outcome of carbapenem monotherapy in the adjunctive treatment of intra-abdominal infections. *Am J Surg* 1997; **174**: 284–290.

Chang Y, Fu H, Xiao Y, Liu J. Nasogastric or nasojejunal feeding in predicted severe acute pancreatitis: a meta-analysis. *Crit Care* 2013; **17**: R118.

Chavez-Tapia NC, Barrientos-Gutierrez T, Tellez-Avila FI, Soares-Weiser K, Uribe M. Antibiotic prophylaxis for cirrhotic patients with upper gastrointestinal bleeding. *Cochrane Database Syst Rev* 2010; (9)CD002907.

Cirocchi R, Trastulli S, Desiderio J, Boselli C, Parisi A, Noya G *et al.* Minimally invasive necrosectomy *versus* conventional surgery in the treatment of infected pancreatic necrosis: a systematic review and a meta-analysis of comparative studies. *Surg Laparosc Endosc Percutan Tech* 2013; **23**: 8–20.

Combier E, Levacher S, Letoumelin P, Joseph A, Pourriat JL, De PG. Cost-effectiveness analysis of the terlipressin–glycerin trinitrate combination in the pre-hospital management of acute gastro-intestinal haemorrhage in cirrhotic patients. *Intensive Care Med* 1999; **25**: 364–370.

de Vries AC, Besselink MG, Buskens E, Ridwan BU, Schipper M, van Erpecum KJ *et al.* Randomized controlled trials of antibiotic prophylaxis in severe acute pancreatitis: relationship between methodological quality and outcome. *Pancreatology* 2007; **7**: 531–538.

Dong Z, Petrov MS, Xu J, Shanbhag S, Windsor JA, Pang S. Peritoneal lavage for severe acute pancreatitis: a systematic review of randomised trials. *World J Surg* 2010; **34**: 2103–2108.

Falagas ME, Matthaiou DK, Bliziotis IA. Systematic review: fluoroquinolones for the treatment of intra-abdominal surgical infections. *Aliment Pharmacol Ther* 2007; **25**: 123–131.

Gaitán HG, Reveiz L, Farquhar C. Laparoscopy for the management of acute lower abdominal pain in women of childbearing age. *Cochrane Database Syst Rev* 2011; (1)CD007683.

Glazer ES, Hornbrook MC, Krouse RS. A meta-analysis of randomized trials: immediate stent placement *vs* surgical bypass in the palliative management of malignant biliary obstruction. *J Pain Symptom Manag* 2014; **47**: 307–314.

Gluud LL, Klingenberg SL, Langholz E. Tranexamic acid for upper gastrointestinal bleeding. *Cochrane Database Syst Rev* 2012; (1)CD006640.

Go JT, Vaughan-Sarrazin M, Auerbach A, Schnipper J, Wetterneck TB, Gonzalez D *et al.* Do hospitalists affect clinical outcomes and efficiency for patients with acute upper gastrointestinal hemorrhage (UGIH)? *J Hosp Med* 2010; **5**: 133–139.

Golub R, Siddiqi F, Pohl D. Role of antibiotics in acute pancreatitis: a meta-analysis. *J Gastrointest Surg* 1998; **2**: 496–503.

Greenwald BD, Caldwell SH, Hespenheide EE, Patrie JT, Williams J, Binmoeller KF *et al.* *N*-2-butyl-cyanoacrylate for bleeding gastric varices: a United States pilot study and cost analysis. *Am J Gastroenterol* 2003; **98**: 1982–1988.

Gregor JC, Ponich TP, Detsky AS. Should ERCP be routine after an episode of ‘idiopathic’ pancreatitis? A cost–utility analysis. *Gastrointest Endosc* 1996; **44**: 118–123.

Haghshenasskashani A, Laurence JM, Kwan V, Johnston E, Hollands MJ, Richardson AJ *et al.* Endoscopic necrosectomy of pancreatic necrosis: a systematic review. *Surg Endosc* 2011; **25**: 3724–3730.

Hamada T, Yasunaga H, Nakai Y, Isayama H, Horiguchi H, Matsuda S *et al.* Continuous regional arterial infusion for acute pancreatitis: a propensity score analysis using a nationwide administrative database. *Crit Care* 2013; **17**: R214.

Hart PA, Bechtold ML, Marshall JB, Choudhary A, Puli SR, Roy PK. Prophylactic antibiotics in necrotizing pancreatitis: a meta-analysis. *South Med J* 2008; **101**: 1126–1131.

Haydock MD, Mittal A, Wilms HR, Phillips A, Petrov MS, Windsor JA. Fluid therapy in acute pancreatitis: anybody’s guess. *Ann Surg* 2013; **257**: 182–188.

Hong WD, Chen XW, Wu WZ, Zhu QH, Chen XR. Metal *versus* plastic stents for malignant biliary obstruction: an update meta-analysis. *Clin Res Hepatol Gastroenterol* 2013; **37**: 496–500.

Hooijmans CR, de Vries RB, Rovers MM, Gooszen HG, Ritskes-Hoitinga M. The effects of probiotic supplementation on experimental acute pancreatitis: a systematic review and meta-analysis. *PLoS One* 2012; **7**: e48811.

Hosono S, Ohtani H, Arimoto Y, Kanamiya Y. Endoscopic stenting *versus* surgical gastroenterostomy for palliation of malignant gastroduodenal obstruction: a meta-analysis. *J Gastroenterol* 2007; **42**: 283–290.

Jafri NS, Mahid SS, Idstein SR, Hornung CA, Galandiuk S. Antibiotic prophylaxis is not protective in severe acute pancreatitis: a systematic review and meta-analysis. *Am J Surg* 2009; **197**: 806–813.

Jairath V, Hearnshaw S, Brunskill SJ, Doree C, Hopewell S, Hyde C *et al.* Red cell transfusion for the management of upper gastrointestinal haemorrhage. *Cochrane Database Syst Rev* 2010; (9)CD006613.

Jiang K, Chen XZ, Xia Q, Tang WF, Wang L. Early nasogastric enteral nutrition for severe acute pancreatitis: a systematic review. *World J Gastroenterol* 2007; **13**: 5253–5260.

Jiang K, Chen XZ, Xia Q, Tang WF, Wang L. Cost-effectiveness analysis of early veno-venous hemofiltration for severe acute pancreatitis in China. *World J Gastroenterol* 2008; **14**: 1872–1877.

Kramer KM, Levy H. Prophylactic antibiotics for severe acute pancreatitis: the beginning of an era. *Pharmacotherapy* 1999; **19**: 592–602.

Lamme B, Boermeester MA, Reitsma JB, Mahler CW, Obertop H, Gouma DJ. Meta-analysis of relaparotomy for secondary peritonitis. *Br J Surg* 2002; **89**: 1516–1524.

Lee JG, Turnipseed S, Romano PS, Vigil H, Azari R, Melnikoff N *et al.* Endoscopy-based triage significantly reduces hospitalization rates and costs of treating upper GI bleeding: a randomized controlled trial. *Gastrointest Endosc* 1999; **50**: 755–761.

Li J, Chen TR, Gong HL, Wan MH, Chen GY, Tang WF. Intensive insulin therapy in severe acute pancreatitis: a meta-analysis and systematic review. *West Indian Med J* 2012; **61**: 574–579.

Li JY, Yu T, Chen GC, Yuan YH, Zhong W, Zhao LN *et al.* Enteral nutrition within 48 hours of admission improves clinical outcomes of acute pancreatitis by reducing complications: a meta- analysis. *PLoS One* 2013; **8**: e64926.

Lingnau W, Berger J, Javorsky F, Lejeune P, Mutz N, Benzer H. Selective intestinal decontamination in multiple trauma patients: prospective, controlled trial. *J Trauma* 1997; **42**: 687–694.

Louie BE, Noseworthy T, Hailey D, Gramlich LM, Jacobs P, Warnock GL. Enteral or parenteral nutrition for severe pancreatitis: a randomized controlled trial and health technology assessment. *Can J Surg* 2005; **48**: 298–306.

Mahar AL, Brar SS, Coburn NG, Law C, Helyer LK. Surgical management of gastric perforation in the setting of gastric cancer. *Gastric Cancer* 2012; **15**(Suppl 1): S146–S152.

Marik PE, Zaloga GP. Meta-analysis of parenteral nutrition *versus* enteral nutrition in patients with acute pancreatitis. *BMJ* 2004; **328**: 1407.

Mazaki T, Ishii Y, Takayama T. Meta-analysis of prophylactic antibiotic use in acute necrotizing pancreatitis. *Br J Surg* 2006; **93**: 674–684.

McClave SA, Chang WK, Dhaliwal R, Heyland DK. Nutrition support in acute pancreatitis: a systematic review of the literature. *J Parenter Enteral Nutr* 2006; **30**: 143–156.

Meng W, Yuan J, Zhang C, Bai Z, Zhou W, Yan J, Li X. Parenteral analgesics for pain relief in acute pancreatitis: a systematic review. *Pancreatology* 2013; **13**: 201–206.

Meng WB, Li X, Li YM, Zhou WC, Zhu XL. Three initial diets for management of mild acute pancreatitis: a meta-analysis. *World J Gastroenterol* 2011; **17**: 4235–4241.

Mofidi R, Suttie SA, Patil PV, Ogston S, Parks RW. The value of procalcitonin at predicting the severity of acute pancreatitis and development of infected pancreatic necrosis: systematic review. *Surgery* 2009; **146**: 72–81.

Morimoto T, Noguchi Y, Sakai T, Shimbo T, Fukui T. Acute pancreatitis and the role of histamine-2 receptor antagonists: a meta-analysis of randomized controlled trials of cimetidine. *Eur J Gastroenterol Hepatol* 2002; **14**: 679–686.

Mouli VP, Sreenivas V, Garg PK. Efficacy of conservative treatment, without necrosectomy, for infected pancreatic necrosis: a systematic review and meta-analysis. *Gastroenterology* 2013; **144**: 333–340.e332.

Nelson RL, Singer M. Primary repair for penetrating colon injuries. *Cochrane Database Syst Rev* 2003; (3)CD002247.

Ockenga J, Borchert K, Rifai K, Manns MP, Bischoff SC. Effect of glutamine-enriched total parenteral nutrition in patients with acute pancreatitis. *Clin Nutr* 2002; **21**: 409–416.

Ofman JJ, MacLean CH, Straus WL, Morton SC, Berger ML, Roth EA *et al.* A metaanalysis of severe upper gastrointestinal complications of nonsteroidal antiinflammatory drugs. *J Rheumatol* 2002; **29**: 804–812.

Papachristou EA, Mitselou MF, Finokaliotis ND. Surgical outcome and hospital cost analyses of laparoscopic and open tension-free hernia repair. *Hernia* 2002; **6**: 68–72.

Petrov MS, Atduev VA, Zagainov VE. Advanced enteral therapy in acute pancreatitis: is there a room for immunonutrition? A meta-analysis. *Int J Surg* 2008; **6**: 119–124.

Petrov MS, Pylypchuk RD, Emelyanov NV. Systematic review: nutritional support in acute pancreatitis. *Aliment Pharmacol Ther* 2008; **28**: 704–712.

Petrov MS, Pylypchuk RD, Uchugina AF. A systematic review on the timing of artificial nutrition in acute pancreatitis. *Br J Nutr* 2009; **101**: 787–793.

Petrov MS, van Santvoort HC, Besselink MG, van der Heijden GJ, Windsor JA, Gooszen HG. Enteral nutrition and the risk of mortality and infectious complications in patients with severe acute pancreatitis: a meta-analysis of randomized trials. *Arch Surg* 2008; **143**: 1111–1117.

Petrov MS, Whelan K. Comparison of complications attributable to enteral and parenteral nutrition in predicted severe acute pancreatitis: a systematic review and meta-analysis. *Br J Nutr* 2010; **103**: 1287–1295.

Petrov MS, Zagainov VE. Influence of enteral *versus* parenteral nutrition on blood glucose control in acute pancreatitis: a systematic review. *Clin Nutr* 2007; **26**: 514–523.

Purkayastha S, Chow A, Athanasiou T, Cambaroudis A, Panesar S, Kinross J *et al.* Does serum procalcitonin have a role in evaluating the severity of acute pancreatitis: a question revisited. *World J Surg* 2006; **30**: 1713–1721.

Seta T, Noguchi Y, Shimada T, Shikata S, Fukui T. Treatment of acute pancreatitis with protease inhibitors: a meta-analysis. *Eur J Gastroenterol Hepatol* 2004; **16**: 1287–1293.

Shafiq N, Malhotra S, Bhasin DK, Rana S, Siddhu S, Pandhi P. Estimating the diagnostic accuracy of procalcitonin as a marker of the severity of acute pancreatitis: a meta-analytic approach. *J Pancreas* 2005; **6**: 231–237.

Sharma VK, Howden CW. Prophylactic antibiotic administration reduces sepsis and mortality in acute necrotizing pancreatitis: a meta-analysis. *Pancreas* 2001; **22**: 28–31.

Sher ME, Weiss EG, Nogueras JJ, Wexner SD. Morbidity of medical therapy for ulcerative colitis: what are we really saving? *Int J Colorectal Dis* 1996; **11**: 287–293.

Singer MA, Nelson RL. Primary repair of penetrating colon injuries: a systematic review. *Dis Colon Rectum* 2002; **45**: 1579–1587.

Singh A, Chen M, Li T, Yang XL, Li JZ, Gong JP. Parenteral nutrition combined with enteral nutrition for severe acute pancreatitis. *ISRN Gastroenterol* 2012; **2012**: 791383.

Sitter H, Lorenz W, Nicolay U, Krack W, Hellenbrandt A, Zielke A *et al.* From clinical evidence to everyday practice: implementing findings from a cost-effectiveness analysis for endoscopic injection therapy for upper-gastrointestinal bleeding. *Eur J Gastroenterol Hepatol* 2003; **13**: 295–304.

Sreedharan A, Martin J, Leontiadis GI, Dorward S, Howden CW, Forman D *et al.* Proton pump inhibitor treatment initiated prior to endoscopic diagnosis in upper gastrointestinal bleeding. *Cochrane Database Syst Rev* 2010; (7)CD005415.

Sun S, Yang K, He X, Tian J, Ma B, Jiang L. Probiotics in patients with severe acute pancreatitis: a meta-analysis. *Langenbecks Arch Surg* 2009; **394**: 171–177.

Theivanayagam S, Lim RG, Cobell WJ, Gowda JT, Matteson ML, Choudhary A *et al.* Administration of erythromycin before endoscopy in upper gastrointestinal bleeding: a meta-analysis of randomized controlled trials. *Saudi J Gastroenterol* 2013; **19**: 205–210.

Tobin K, Klein J, Barbieri C, Heffner JE. Utility of routine admission chest radiographs in patients with acute gastrointestinal haemorrhage admitted to an intensive care unit. *Am J Med* 1996; **101**: 349–356.

van Baal MC, van Santvoort HC, Bollen TL, Bakker OJ, Besselink MG, Gooszen HG; Dutch Pancreatitis Study Group. Systematic review of percutaneous catheter drainage as primary treatment for necrotizing pancreatitis. *Br J Surg* 2011; **98**: 18–27.

van Brunschot S, Fockens P, Bakker OJ, Besselink MG, Voermans RP, Poley JW *et al.* Endoscopic transluminal necrosectomy in necrotising pancreatitis: a systematic review. *Surg Endosc* 2014: **28**: 1425–1438.

van Ruler O, Mahler CW, Boer KR, Reuland EA, Gooszen HG, Opmeer BC *et al.*; Dutch Peritonitis Study Group. Comparison of on-demand vs planned relaparotomy strategy in patients with severe peritonitis: a randomized trial. *JAMA* 2007; **298**: 865–872.

Wang J, Bao YX, Bai M, Zhang YG, Xu WD, Qi XS. Restrictive *vs* liberal transfusion for upper gastrointestinal bleeding: a meta-analysis of randomized controlled trials. *World J Gastroenterol* 2013; **19**: 6919–6927.

Wang Q, Guo Z, Zhao P, Wang Y, Gan T, Yang J. Chinese herbal medicines for acute pancreatitis. *Cochrane Database of Syst Rev* 2005; (1)CD003631.

Wells M, Chande N, Adams P, Beaton M, Levstik M, Boyce E *et al.* Meta-analysis: vasoactive medications for the management of acute variceal bleeds. *Aliment Pharmacol Ther* 2012; **35**: 1267–1278.

Winstead NS, Wilcox CM. Erythromycin prior to endoscopy for acute upper gastrointestinal haemorrhage: a cost-effectiveness analysis. *Aliment Pharmacol Ther* 2007; **26**: 1371–1377.

Wu LM, Xu JR, Yin Y, Qu XH. Usefulness of CT angiography in diagnosing acute gastrointestinal bleeding: a meta-analysis. *World J Gastroenterol* 2010; **16**: 3957–3963.

Xu T, Cai Q. Prophylactic antibiotic treatment in acute necrotizing pancreatitis: results from a meta-analysis. *Scand J Gastroenterol* 2008; **43**: 1249–1258.

Xu W, Zhou YF, Xia SH. Octreotide for primary moderate to severe acute pancreatitis: a meta-analysis. *Hepatogastroenterology* 2013; **60**: 1504–1508.

Yang C, Guanghua F, Wei Z, Zhong J, Penghui J, Xin F *et al.* Combination of hemofiltration and peritoneal dialysis in the treatment of severe acute pancreatitis. *Pancreas* 2010; **39**: 16–19.

Yao L, Huang X, Li Y, Shi R, Zhang G. Prophylactic antibiotics reduce pancreatic necrosis in acute necrotizing pancreatitis: a meta-analysis of randomized trials. *Dig Surg* 2010; **27**: 442–449.

Yasunaga H, Horiguchi H, Hashimoto H, Matsuda S, Fushimi K. Effect and cost of treatment for acute pancreatitis with or without gabexate mesylate: a propensity score analysis using a nationwide administrative database. *Pancreas* 2013; **42**: 260–264.

Yi F, Ge L, Zhao J, Lei Y, Zhou F, Chen Z *et al.* Meta-analysis: total parenteral nutrition *versus* total enteral nutrition in predicted severe acute pancreatitis. *Intern Med* 2012; **51**: 523–530.

Zhang MM, Cheng JQ, Lu YR, Yi ZH, Yang P, Wu XT. Use of pre-, pro- and synbiotics in patients with acute pancreatitis: a meta-analysis. *World J Gastroenterol* 2010; **16**: 3970–3978.

Zhou WC, Li YM, Zhang H, Li X, Zhang L, Meng WB *et al.* Therapeutic effects of endoscopic therapy combined with enteral nutrition on acute severe biliary pancreatitis. *Chin Med J* 2011; **124**: 2993–2996.

**Not an eligible intervention**

Al-Omran M, AlBalawi ZH, Tashkandi MF, Al-Ansary LA. Enteral *versus* parenteral nutrition for acute pancreatitis. *Cochrane Database Syst Rev* 2010; (1)CD002837.

Andersen BR, Kallehave FL, Andersen HK. Antibiotics *versus* placebo for prevention of postoperative infection after appendicectomy. *Cochrane Database Syst Rev* 2005; (3)CD001439.

Andriulli A, Annese V, Caruso N, Pilotto A, Accadia L, Niro AG *et al.* Proton-pump inhibitors and outcome of endoscopic hemostasis in bleeding peptic ulcers: a series of meta-analyses. *Am J Gastroenterol* 2005; **100**: 207–219.

Bai Y, Guo JF, Li ZS. Meta-analysis: erythromycin before endoscopy for acute upper gastrointestinal bleeding. *Aliment Pharmacol Ther* 2011; **34**: 166–171.

Bardou M, Toubouti Y, Benhaberou-Brun D, Rahme E, Barkun AN. Meta-analysis: proton-pump inhibition in high-risk patients with acute peptic ulcer bleeding. *Aliment Pharmacol Ther* 2005; **21**: 677–686.

Barkun AN, Bardou M, Martel M, Gralnek IM, Sung JJ. Prokinetics in acute upper GI bleeding: a meta-analysis. *Gastrointest Endosc* 2010; **72**: 1138–1145.

Barkun AN, Martel M, Toubouti Y, Rahme E, Bardou M. Endoscopic hemostasis in peptic ulcer bleeding for patients with high-risk lesions: a series of meta-analyses. *Gastrointest Endosc* 2009; **69**: 786–799.

Bustamante M, Stollman N. The efficacy of proton-pump inhibitors in acute ulcer bleeding: a qualitative review. *J Clin Gastroenterol* 2000; **30**: 7–13.

García-López S, Gomollón-García F, Pérez-Gisbert J. Cyclosporine in the treatment of severe attack of ulcerative colitis: a systematic review. *Gastroenterol Hepatol* 2005; **28**: 607–614.

Gisbert JP, González L, Calvet X, Roqué M, Gabriel R, Pajares JM. Proton pump inhibitors *versus* H2-antagonists: a meta-analysis of their efficacy in treating bleeding peptic ulcer. *Aliment Pharmacol Ther* 2001; **15**: 917–926.

Imperiale TF, Birgisson S. Somatostatin or octreotide compared with H2 antagonists and placebo in the management of acute nonvariceal upper gastrointestinal hemorrhage: a meta-analysis. *Ann Intern Med* 1997; **127**: 1062–1071.

Kahi CJ, Jensen DM, Sung JJ, Bleau BL, Jung HK, Eckert G *et al.* Endoscopic therapy *versus* medical therapy for bleeding peptic ulcer with adherent clot: a meta-analysis. *Gastroenterology* 2005; **129**: 855–862.

Khuroo MS, Khuroo MS, Farahat KL, Kagevi IE. Treatment with proton pump inhibitors in acute non-variceal upper gastrointestinal bleeding: a meta-analysis. *J Gastroenterol Hepatol* 2005; **20**: 11–25.

Laine L, McQuaid KR. Endoscopic therapy for bleeding ulcers: an evidence-based approach based on meta-analyses of randomized controlled trials. *Clin Gastroenterol Hepatol* 2009; **7**: 33–47.

Leontiadis GI, Sharma VK, Howden CW. Systematic review and meta-analysis of proton pump inhibitor therapy in peptic ulcer bleeding. *BMJ* 2005; **330**: 568–570.

Leontiadis GI, Sreedharan A, Dorward S, Barton P, Delaney B, Howden CW *et al.* Systematic reviews of the clinical effectiveness and cost-effectiveness of proton pump inhibitors in acute upper gastrointestinal bleeding. *Health Technol Assess* 2007; **11**: 1–164.

Leontiadis GI, Sreedharan A, Dorward S, Barton P, Delaney B, Howden CW *et al.* Systematic reviews of the clinical effectiveness and cost-effectiveness of proton pump inhibitors in acute upper gastrointestinal bleeding: PPI therapy in patients with endoscopically documented acute bleeding from a peptic ulcer. *Health Technol Assess* 2007; **11**: 15–39.

Leontiadis GI, Sreedharan A, Dorward S, Barton P, Delaney B, Howden CW *et al.* Systematic reviews of the clinical effectiveness and cost-effectiveness of proton pump inhibitors in acute upper gastrointestinal bleeding: PPI therapy initiated prior to endoscopy. *Health Technol Assess* 2007; **11**: 41–51.

Lin PC, Chang CH, Hsu PI, Tseng PL, Huang YB. The efficacy and safety of proton pump inhibitors *vs* histamine-2 receptor antagonists for stress ulcer bleeding prophylaxis among critical care patients: a meta-analysis. *Crit Care Med* 2010; **38**: 1197–1205.

Marmo R, Rotondano G, Piscopo R, Bianco MA, D'Angella R, Cipolletta L. Dual therapy *versus* monotherapy in the endoscopic treatment of high-risk bleeding ulcers: a meta-analysis of controlled trials. *Am J Gastroenterol* 2007; **102**: 279–289.

Neumann I, Letelier Luz M, Rada G, Claro Juan C, Martin J, Howden Colin W *et al.* Comparison of different regimens of proton pump inhibitors for acute peptic ulcer bleeding. *Cochrane Database Syst Rev* 2013; (6)CD007999.

Selby NM, Kubba AK, Hawkey CJ. Acid suppression in peptic ulcer haemorrhage: a ‘meta-analysis’. *Aliment Pharmacol Ther* 2000; **14**: 1119–1126.

Spiegel BM, Vakil NB, Ofman JJ. Endoscopy for acute nonvariceal upper gastrointestinal tract hemorrhage: is sooner better? A systematic review. *Arch Intern Med* 2001; **161**: 1393–1402.

Sung JJ, Tsoi KK, Lai LH, Wu JC, Lau JY. Endoscopic clipping *versus* injection and thermo-coagulation in the treatment of non-variceal upper gastrointestinal bleeding: a meta-analysis. *Gut* 2007; **56**: 1364–1372.

Tomtitchong P, Siribumrungwong B, Vilaichone RK, Kasetsuwan P, Matsukura N, Chaiyakunapruk N. Systematic review and meta-analysis: *Helicobacter pylori* eradication therapy after simple closure of perforated duodenal ulcer. *Helicobacter* 2012; **17**: 148–152.

Tsoi KK, Chan HC, Chiu PW, Pau CY, Lau JY, Sung JJ. Second-look endoscopy with thermal coagulation or injections for peptic ulcer bleeding: a meta-analysis. *J Gastroenterol Hepatol* 2010; **25**: 8–13.

Tsoi KK, Hirai HW, Sung JJ. Meta-analysis: comparison of oral *vs.* intravenous proton pump inhibitors in patients with peptic ulcer bleeding. *Aliment Pharmacol Ther* 2013; **38**: 721–728.

Vergara M, Calvet X, Gisbert JP. Epinephrine injection *versus* epinephrine injection and a second endoscopic method in high risk bleeding ulcers. *Cochrane Database Syst Rev* 2007; (2)CD005584.

Wang CH, Ma MH, Chou HC, Yen ZS, Yang CW, Fang CC *et al.* High-dose *vs* non-high-dose proton pump inhibitors after endoscopic treatment in patients with bleeding peptic ulcer: a systematic review and meta-analysis of randomized controlled trials. *Arch Intern Med* 2010; **170**: 751–758.

Wang J, Yang K, Ma B, Tian J, Liu Y, Bai Z *et al.* Intravenous pantoprazole as an adjuvant therapy following successful endoscopic treatment for peptic ulcer bleeding. *Can J Gastroenterol* 2009; **23**: 287–299.

Wittau M, Mayer B, Scheele J, Henne-Bruns D, Dellinger EP, Isenmann R. Systematic review and meta-analysis of antibiotic prophylaxis in severe acute pancreatitis. *Scand J Gastroenterol* 2011; **46**: 261–270.

Wu LC, Cao YF, Huang JH, Liao C, Gao F. High-dose *vs* low-dose proton pump inhibitors for upper gastrointestinal bleeding: a meta-analysis. *World J Gastroenterol* 2010; **16**: 2558–2565.

Yuan Y, Wang C, Hunt RH. Endoscopic clipping for acute nonvariceal upper-GI bleeding: a meta-analysis and critical appraisal of randomized controlled trials. *Gastrointest Endosc* 2008; **68**: 339–351.

Zed PJ, Loewen PS, Slavik RS, Marra CA. Meta-analysis of proton pump inhibitors in treatment of bleeding peptic ulcers. *Ann Pharmacother* 2001; **35**: 1528–1534.

**Not in adults (paediatric)**

Bucher BT, Hall BL, Warner BW, Keller MS. Intussusception in children: cost-effectiveness of ultrasound vs diagnostic contrast enema. *J Pediatr Surg* 2011; **46**: 1099–1105.

Ciarrocchi A, Amicucci G. Safety and impact on diagnostic accuracy of early analgesia in suspected acute appendicitis: a meta-analysis. *Int J Surg* 2013; **11**: 847–852.

Firilas AM, Higginbotham PH, Johnson DD, Jackson RJ, Wagner CW, Smith SD. A new economic benchmark for surgical treatment of appendicitis. *Am Surg* 1999; **65**: 769–773.

Peña BMG, Taylor GA, Fishman SJ, Mandl KD. Costs and effectiveness of ultrasonography and limited computed tomography for diagnosing appendicitis in children. *Pediatrics* 2000; **106**: 672–676.

Myers AL, Williams RF, Giles K, Waters TM, Eubanks JW III, Hixson SD *et al.* Hospital cost analysis of a prospective, randomized trial of early *vs* interval appendectomy for perforated appendicitis in children. *J Am Coll Surg* 2012; **214**: 427–434.

Rao SC, Basani L, Simmer K, Samnakay N, Deshpande G. Peritoneal drainage *versus* laparotomy as initial surgical treatment for perforated necrotizing enterocolitis or spontaneous intestinal perforation in preterm low birth weight infants. *Cochrane Database Syst Rev* 2011; (6)CD006182.

Weiner DJ, Katz A, Hirschl RB, Drongowski R, Coran AG. Interval appendectomy in perforated appendicitis. *Pediatr Surg Int* 1995; **10**: 82–85.

**Review protocols**

Alhazzani W, Win LL, Howden CW, Leontiadis GI. Somatostatin or somatostatin analogues for acute non-variceal upper gastrointestinal bleeding. *Cochrane Database Syst Rev* 2011; (10).CD009381.

Gurusamy KS, Kumar Y, Farouk M, Davidson BR. Methods of management of high-risk surgical patients with acute cholecystitis. *Cochrane Database Syst Rev* 2008; (2)CD007088.

Kukuruzovic RH, Chauhan S, Dorney S, Elliott EJ. Antibiotics for cholangitis and/or cholecystitis. *Cochrane Database Syst Rev* 2002; (3)CD003780.

Liu L, Hua F, Li Q, Wang K, Shao J. Surgery for complicated diverticular disease: primary or secondary anastomosis after colonic resection. *Cochrane Database Syst Rev* 2011; (12)CD006141.

Peng S, Cheng Y, Zhang Y, Zhou J, Liao Y, Cheng N *et al.* Appendix stump closure during laparoscopic appendectomy. *Cochrane Database Syst Rev* 2012; (12)CD006437.

Shabanzadeh DM. Antibiotics for uncomplicated acute diverticulitis. *Cochrane Database Syst Rev* 2011; (4)CD009092.

Wild JRL, Abdul N, Ritchie JE, Rud B, Freels S, Nelson RL. Ultrasonography for diagnosis of acute appendicitis. *Cochrane Database Syst Rev* 2013; (2)CD010402.

**Publication withdrawn or retracted**

Havanond C, Havanond P. Argon plasma coagulation therapy for acute non-variceal upper gastrointestinal bleeding. *Cochrane Database Syst Rev* 2009; (4)CD003791. [Withdrawn]

Leontiadis GI, Sharma VK, Howden CW. Proton pump inhibitor treatment for acute peptic ulcer bleeding. *Cochrane Database Syst Rev* 2010; (5)CD002094. [Withdrawn]

Siddiqui A, Khandelwal N, Anthony T, Huerta S. Colonic stent *versus* surgery for the management of acute malignant colonic obstruction: a decision analysis. *Aliment Pharmacol Ther* 2007; **26**: 1379–1386. [Retracted]

**Duplicate publications of the same review or older reviews for which updated version was available and included**

Gluud LL, Klingenberg SL, Langholz SE. Systematic review: tranexamic acid for upper gastrointestinal bleeding. *Aliment Pharmacol Ther* 2008; **27**: 752–758. [Duplicate publication to a Cochrane review of the same title and authors]

Gurusamy KS, Samraj K. Early *versus* delayed laparoscopic cholecystectomy for acute cholecystitis. *Cochrane Database Syst Rev* 2006; (4)CD005440. [Updated version included]

Leontiadis GI, Sreedharan A, Dorward S, Barton P, Delaney B, Howden CW *et al.* Systematic reviews of the clinical effectiveness and cost-effectiveness of proton pump inhibitors in acute upper gastrointestinal bleeding. *Health Technol Assess* 2007; **11**: i–xi, 1. [Executive summary of the full report Leontiadis HTA 11(51)]

Sanabria A, Villegas MI, Morales Uribe CH. Laparoscopic repair for perforated peptic ulcer disease. *Cochrane Database Syst Rev* 2005; (4)CD004778. [Updated version included]

**Other reasons**

Borzellino G, Sauerland S, Minicozzi AM, Verlato G, Di Pietrantonj C, de Manzoni G *et al.* Laparoscopic cholecystectomy for severe acute cholecystitis: a meta-analysis of results. *Surg Endosc* 2008; **22**: 8–15. [Comparison between patients with different disease severity rather than interventions]

Jhee SS, Gill MA, Yellin AE, Berne TV, Heseltine PN, Appleman MD. Pharmacoeconomics of piperacillin/tazobactam and imipenem/cilastatin in the treatment of patients with intra-abdominal infections. *Clin Ther* 1995; **17**: 126–135. [Not focused on the ‘main’ intervention for treatment of eligible condition]

Spiegel BM, Ofman JJ, Woods K, Vakil NB. Minimizing recurrent peptic ulcer haemorrhage after endoscopic haemostasis: the cost-effectiveness of competing strategies. *Am J Gastroenterol* 2003; **98**: 86–97. [Focus is on follow-up procedures after the main intervention to stop bleeding]
